# Efficient Electrooxidation of 5‐Hydroxymethylfurfural Using Co‐Doped Ni_3_S_2_ Catalyst: Promising for H_2_ Production under Industrial‐Level Current Density

**DOI:** 10.1002/advs.202200957

**Published:** 2022-04-15

**Authors:** Yan Sun, Jie Wang, Yufeng Qi, Wenjiang Li, Cheng Wang

**Affiliations:** ^1^ Tianjin Key Laboratory of Advanced Functional Porous Materials Institute for New Energy Materials & Low‐Carbon Technologies School of Materials Science and Engineering Tianjin University of Technology Tianjin 300384 P. R. China; ^2^ Key Laboratory of Display Materials & Photoelectric Devices School of Materials Science and Engineering Tianjin University of Technology Tianjin 300384 P. R. China

**Keywords:** 5‐hydroxymethylfurfural, Co‐doping, large current density, phase reconstruction

## Abstract

Replacing oxygen evolution reaction (OER) by electrooxidations of organic compounds has been considered as a promising approach to enhance the energy conversion efficiency of the electrolytic water splitting proces. Developing efficient electrocatalysts with low potentials and high current densities is crucial for the large‐scale productions of H_2_ and other value‐added chemicals. Herein, non‐noble metal electrocatalysts Co‐doped Ni_3_S_2_ self‐supported on a Ni foam (NF) substrate are prepared and used as catalysts for 5‐hydroxymethylfurfural (HMF) oxidation reaction (HMFOR) under alkaline aqueous conditions. For HMFOR, the Co_0.4_NiS@NF electode achieves an extremely low onset potential of 0.9 V versus reversible hydrogen electrode (RHE) and records a large current density of 497 mA cm^–2^ at 1.45 V versus RHE for HMFOR. During the HMFOR‐assisted H_2_ production, the yield rates of 2,5‐furandicarboxylic acid (FDCA) and H_2_ in a 10 mL electrolyte containing 10 × 10^−3^ M HMF are 330.4 µmol cm^–2^ h^–1^ and 1000 µmol cm^–2^ h^–1^, respectively. The Co_0.4_NiS@NF electrocatalyst displays a good cycling durability toward HMFOR and can be used for the electrooxidation of other biomass‐derived chemicals. The findings present a facile route based on heteroatom doping to fabricate high‐performance catalyses that can facilitate the industrial‐level H_2_ production by coupling the conventional HER cathodic processes with HMFOR.

## Graphical abstract

A facile route based on in‐situ heteroatom doping was developed to fabricate Co‐doped Ni_3_S_2_ electrocatalysts on Ni foam (NF) substrates. The optimized electrocatalyst (Co_0.4_NiS@NF) electrode requires an extremely low onset potential and records a high current density for the 5‐hydroxymethylfurfural (HMF) oxidation reaction (HMFOR) and facilitates the industrial‐level H_2_ production by coupling the conventional HER cathodic process with HMFOR.

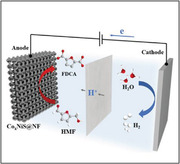

## 1 Introduction

The increasing global energy demand and environmental crisis posed by the contiuned use of fossil fuels necessitate the development of sustainable and clean energy storage and conversion technologies. Hydrogen (H_2_) has been considered as an attractive alternative to conventional fossilized resources due to its high energy density, nontoxicity, and near‐zero greenhouse gas emissions.^[^ ^1^ ^]^ According to the International Energy Agency, the projected annual global industrial H_2_ production in 2070 is expected to reach 520 million tons. Relative to the conventional H_2_ production technologies that use fossilized resources, water electrolysis driven by renewable energy is a sustainable route that allows the production of high purity H_2_ (>99.9%) with no carbon emissions.^[^ ^2^ ^]^ In industrial processes, the electrocatalytic hydrogen evolution reaction (HER) has been mainly achieved in the cost‐efficiency alkaline aqueous media due to the highly corrosive nature of acidic solutions. However, the H_2_ production rate in alkaline water splitting is usually two to three orders of magnitude lower than that under acidic conditions due to the low‐efficiency of the proton generation rate and high energy consumption of the anodic oxygen evolutionreaction (OER).^[^ ^3^ ^]^ To improve the production rate, noble metal catalysts are used to facilitate the half reactions. Furthermore, H_2_/O_2_ separators are also used to avoid gas cross over during the water electrolyzer operation. These indispensable device components make the H_2_ production cost (US$ 4 Kg^−1^) through water splitting two or three times higher than that through fossil fuel reforming.^[^ ^2^, ^5^ ^]^

**Scheme 1:**
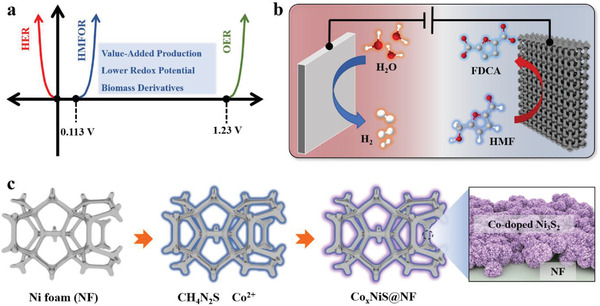
a) *I*–*V* curves and required potentials at the cathode and anode for electrolysis, b) HMF electrooxidation couple with HER for FDCA and H_2_ productions and c) schematic of the synthesis procedure of the Co*_x_* NiS@NF electrocatalysts.

To circumvent these challenges, the alkaline electrooxidation of organic compounds is considered as an alternative to the conventional OER process due to its more favorable thermodynamic considerations and more economical requirements.^[^ ^6^ ^]^ Among numerous oxidizable organic molecules, such as, methanol,^[^ ^7^ ^]^ ethanol,^[^ ^8^ ^]^ benzyl alcohol,^[^ ^9^ ^]^ furfural,^[^ ^10^ ^]^ furfuryl alcohol,^[^ ^11^ ^]^ glucose,^[^ ^12^ ^]^ 5‐hydroxymethylfurfural (HMF),^[^ ^13^ ^]^ biomass derivatives from which high‐added value chemicals can be produced have attracted great industrial and scientific interests due to their low oxidation potentials than OER (**Scheme** **1**a).^[^ ^6^, ^14^, ^15^ ^]^ Biomass is a renewable, earth‐abundant organic material that is formed through the natural conversion of CO_2_ and H_2_O into carbohydrates during photosynthesis. Through Highly efficient conversion routes, these non‐grain carbon resources can be transformed into value‐added chemicals fuels, which are promising alternatives that can reduce CO_2_ carbon dioxide emissions related to the use of fossil fuels. HMF, which is derived from the acid‐catalyzed dehydration of C6 carbohydrates, is among the most commonly used biomass‐based furan compound and has been well known as one of the key bridges connecting nonedible biomass‐based chemicals and fossil‐based chemicals.^[^ ^16^ ^]^ Thestandard electrooxidation potential of HMF (0.113 V) is much lower than the standard potential for OER (1.23 V).^[^ ^17^, ^18^ ^]^ Among the HMF oxidation products, 2,5‐furan dicarboxylic acid (FDCA) can serve as the major biobased alternative precursor to terephthalic acid, one of the petroleum‐derived monomers of various polyesters, for the productions of many commercialized polymers such as petroleum‐derived polyethylene terephthalate (PET) and polybutylene terephthalate (PBT). The global market size of PET and PBT is approximately 18.76 million tons, which correspond to 7.4% and 0.25% of the global plastics production and primary energy consumption, respectively.^[^ ^19^, ^20^ ^]^ Conventionally, FDCA is synthesized via the stoichiometric chemical oxidation of HMF by O_2_ or air at high temperatures (50–160 °C) and pressure (1–40 bar), using noble metal catalysts.^[^ ^21^ ^]^ This process inevitably generates by‐products that necessitate the integration of expensive and energy‐intensive separation processes.^[^ ^22^ ^]^ The high selective electrooxidation of HMF to FDCA is a more facile, benign, and economical method than the traditional routes for the production of a value‐added chemical.^[^ ^23^ ^]^ Moreover, replacing OER with HMF electrooxidation to couple with HER for FDCA and H_2_ productions (Scheme 1b) possesses safer and higher energy efficiency with a nearly 200% combined theoretical faradaic efficiency (FE).^[^ ^2^, ^24^ ^]^ Even though, the competitive OER is regarded as one of the key restrictions that will narrow down the potential range for HMF oxidation, which results to low current densities and charge utilization efficiencies (low FE).^[^ ^25^ ^]^ At high pH conditions,the electrochemical oxidation of HMF is facilitated due to the promotion of charge transfer processes and biased reaction equilibria. However, HMF could easily degrade into unfavorable Humins in an alkaline electrolyte environment.^[^ ^26^ ^]^ To minimize the excessive HMF degradation and achieve an industrial‐level high current density (>400 mA cm^−2^) for large‐scale FDCA and H_2_ productions under favorable potential in alkaline aqueous condition, the mass transfer of HMF should be fast and the activity for HMFOR on electrocatalysts needs to be expedited to shorten the electrolysis time.

The utilization of non‐noble metal‐based electrocatalysts such as oxides, hydroxides, nitrides, phosphides, and sulfides to lower the onset potential of HMFOR has been studied extensively.^[^ ^27^, ^28^ ^]^ Although these catalysts work well at low current densities, their high intrinsic redox potential and/or slower kinetics make them difficult to achieve the higher current densities at the favored potential.^[^ ^29^ ^]^ In order to achieve a high current density, the catalytically active sites of a suitable catalyst should meet several criteria simultaneously. i) High intrinsic activities for HMFOR, ii) high densities on the catalyst's surfaces, iii) faster charge/electron transports and iv) high tolerances against corrosion under strongly oxidizing conditions.^[^ ^4^ ^]^ Ni‐based catalysts is one of the most active non‐noble metal OER electrocatalysts due to their good inherent oxygen affinity and faster reaction kinetics.^[^ ^30^ ^]^ Higher current densities may be attained once the mass transfer limitations are overcome. As common metal sulfide, Ni_3_S_2_ has been identified as a potential catalyst for various electrochemical reactions because of its rich valence states, fast electron transfer rate and abundant active sites.^[^ ^31^, ^32^, ^33^, ^34^ ^]^ Recently, Ni_3_S_2_‐based electodes subjected to defect engineering, heteroatom doping, and advanced structure design exhibited extraordinary performance in the field of water splitting.^[^ ^35^, ^36^, ^37^ ^]^ Among these methodologies, doping metallic elements close to Ni such as Fe,^[^ ^38^ ^]^ Co,^[^ ^39^ ^]^ and Cu^[^ ^40^ ^]^ into nanostructured Ni_3_S_2_ materials is an effective strategy as the incorporated cations can increase the electrochemically active surface area and/or electron transfer. The current research on Ni_3_S_2_‐based materials for water splitting is more in‐depth, the performance in the HMFOR‐assistant hydrogen production system remains to be optimized. Therefore, it is desirable to modulate the electronic structure through the defect and interfacial engineering, element doping, and so on to improve their catalytic activity.

Here, self‐supported Co‐doped Ni_3_S_2_ electrocatalysts were directly grown on Ni foam substrate (denoted as Co*_x_* NiS@NF) to construct 3D porous and binder‐free integrated electrodes. Although Co‐based materials are poor HMFOR electrocatalysts, akin to nickel‐based catalysts,^[^ ^41^, ^42^ ^]^ Co doping not only promoted the formation of nanosheet‐like arrayed Co*_x_* NiS@NF with abundant active sites but also effectively reduced the HMFOR overpotential. The optimized doping product, Co_0.4_NiS@NF, displayed a superior activity for HMF electrooxidation with a lower onset potential (0.9 V vs RHE). To achieve a current density of 10 mA cm^–2^ for HMFOR, only 1.04 V versus RHE was needed, which is reduced by 430 mV than that of OER. Moreover, a large current density of 497 mA cm^–2^ can be reached at 1.45 V versus RHE (without IR compensation). The performance outperforms previously published state‐of‐the‐art Ni/Co‐based electrocatalysts. Coupled with cathodic HER in alkaline media, it delivers the yield rates of 330.4 µmol cm^–2^ h^–1^ for FDCA and 1000 µmol cm^–2^ h^–1^ for H_2_. The effects of electrolysis temperature and HMF concentration on HMFOR were also investigated. Additionally, the performance of the best performing catalyst toward the electrocatalytic oxidation of other biomass‐derived platform substrates, such as glucose, fructose, furfural, and benzoic acid was also studied. This study reports the fabrication of high‐performance catalysts for the potential industrial level HMFOR‐assisted H_2_ production systems.

## 2 Results and Discussion

### 2.1 Characterization of Electrocatalysts

**Figure 1:**
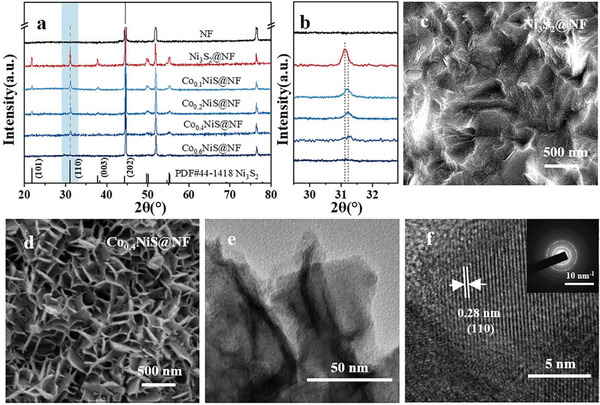
a) XRD patterns of the as‐synthesized Ni_3_S_2_@NF, Co*_x_* NiS@NF, and NF, b) corresponding enlarged XRD patterns. SEM images of c) Ni_3_S_2_@NF and d) Co_0.4_NiS@NF. e) TEM image and f) HRTEM image of Co_0.4_NiS@NF (inset: SAED pattern).

The Co*_x_* NiS@NF were synthesized by a facile one‐step hydrothermal reaction as illustrated in Scheme 1c. During the hydrothermal process, CoCl_2_·6H_2_O and part of the surficial nickel of the NF substrate were converted into their corresponding metal sulfides with the presence of thiourea. The leaching and/or dissolution of Ni from NF as well as soluble Co^2+^ provide metallic sources for the formation of these metal sulfides.^[^ ^43^ ^]^ X‐ray diffraction patterns (XRD) reveal (**Figure** **1**a) that most of the diffraction peaks of the five samples match well with the standard hexagonal Ni_3_S_2_ (PDF# 44‐1418) along with the NF substrate. They are accordingly denoted as Ni_3_S_2_@NF, Co_0.1_NiS@NF, Co_0.2_NiS@NF, Co_0.4_NiS@NF, and Co_0.6_NiS@NF. The Co/Ni ratios in the four Co*_x_* NiS@NF, given by ICP‐AES (Table S1, Supporting Information), are 1/23.5, 1/7.1, 1/3.7, and 1/2.4. These values corresponded well to the increase in the amount of CoCl_2_·6H_2_O added in the hydrothermal precursor solution. Meanwhile, the crystallinity of four Co*_x_* NiS@NF samples decreases with increasing Co content and the metal sulfides in Co_0.6_NiS@NF are nearly amorphous. It was noticed that no precipitate could be yielded from CoCl_2_·6H_2_O and thiourea solution in the absence of NF within our investigated amount range of these two precursors. In addition, neither other crystalline precipitate was formed in the reaction system other than metal sulfides formed on NF substrate. These findings suggest that NF plays a pivotal role in forming Co*_x_* NiS@NF. Despite having similar diffraction patterns, the diffraction peaks of Co_x_NiS@NF slightly shifted to higher angles with increasing Co concentration. Such phenomenon could be evidenced by the shift of the (110) plane of Ni_3_S_2_ in Figure 1b. Therefore, doping Co into the Ni_3_S_2_ host lattice leads to lattice distortions and a decrease in crystallinity.

After hydrothermal sulfidation, the relatively smooth surface (Figure S1, Supporting Information) of NF became roughened first (Figure 1c) and then was gradually covered by Co*_x_* NiS@NF (Figure S2, Supporting Information) with the presence of CoCl_2_·6H_2_O under SEM observations. When *x* is less than 0.4 mmol, the NF surfaces consist of uniform Co*_x_* NiS arrays. The dimensionals of these domains varied from the submicrometer to micrometer scale with increasing *x* (Figure S2b,d,f,h, Supporting Information). Further increasing *x* to 0.6 mmol, discrete larger particles become discernible (Figure S2ij, Supporting Information). Since Co_0.4_NiS@NF exhibited the best electrocatalytic performance, it was used for the following characterizations unless specified. SEM (Figure 1d) and TEM (Figure 1e) show that the microscale domain of Co_0.4_NiS@NF is comprised of interconnected nanosheets with thickness below 40 nm. A lattice spacing of 0.28 nm corresponding to the (110) crystal plane of Ni_3_S_2_ could be observed under HRTEM (Figure 1f) and a set of well‐defined diffraction patterns was observed in SAED pattern (inset in Figure 1f).

**Figure 2:**
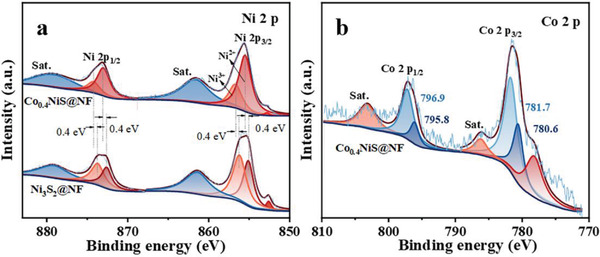
High‐resolution XPS spectra of Ni 2p for a) Co_0.4_NiS@NF and Ni_3_S_2_@NF, and b) Co 2p for Co_0.4_NiS@NF.

The presence of Co, Ni, and S elements in Co_0.4_NiS@NF is confirmed by XPS survey spectra (Figure S3a, Supporting Information). The high‐resolution XPS spectrum of Ni 2p can be fitted into two spin‐orbit doublet peaks, including the binding energies at 855.5 (Ni 2p_3/2_) and 873.0 eV (Ni 2p_1/2_) for Ni^2+^ and 856.7 (Ni 2p_3/2_) and 874.2 eV (Ni 2p_1/2_) for Ni^3+^ (**Figure** **2**a).^[^ ^37^ ^]^ Besides a single peak of metallic Ni at 852.8 eV, there are also two nickel satellite peaks (marked as “Sat.”) at higher binding energies for both Ni 2P_1/2_ and Ni 2p_3/2_.^[^ ^4^, ^44^ ^]^ Compared with those of Ni_3_S_2_@NF, the Ni 2p peaks shift about 0.4 eV to higher binding energies and the amount of metallic Ni is reduced in Co_0.4_NiS@NF (Figure 2a), implying that the Co incorporation might modulate the electronic structure of the Ni center.^[^ ^38^, ^45^ ^]^ Similarly, the Co 2p spectrum of Co_0.4_NiS@NF shows two spin–orbit doublets of Co^3+^ (780.6 eV of Co 2p_3/2_ and 795.8 eV of Co 2p_1/2_) and Co^2+^ (781.7 eV of Co 2p_3/2_ and 796.9 eV Co 2p_1/2_) (Figure 2b).^[^ ^46^ ^]^ As for S 2p spectrum, the peaks at 161.9 and 163.0 eV could be ascribed to S 2p_3/2_ and S 2p_1/2_ of S^2–^ species in Co_0.4_NiS@NF (Figure S3b, Supporting Information).^[^ ^47^ ^]^ Based on the results of XRD and XPS, it can be verified that Co was successfully incorporated into the lattice of Ni_3_S_2_ in Co_0.4_NiS@NF.

### 2.2 OER and HMF Electrooxidation

**Figure 3:**
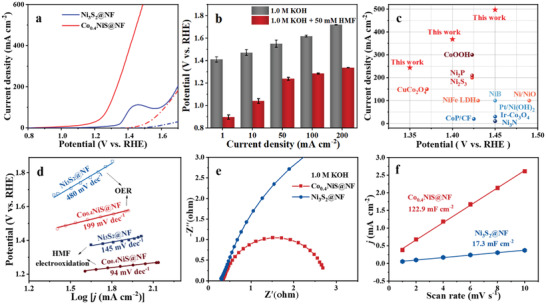
a) LSV curves of the Co_0.4_NiS@NF and Ni_3_S_2_@NF. b) Potentials required for delivering different current densities using the Co_0.4_NiS@NF electrocatalyst (without IR compensation) in 1.0 m KOH aqueous solution with (solid lines) and without (dot‐dash lines) 50 × 10^−3^ m HMF. c) Comparison of the performance of Co_0.4_NiS@NF with those of previously reported electrocatalysts. d) Tafel plots, e) Nyquist plots, and f) the capacitive current densities for HMFOR at 0.65 V under various scan rates of Co_0.4_NiS@NF and Ni_3_S_2_@NF.

The OER and HMF electrooxidation reaction (HMFOR) performances of the synthesized Ni_3_S_2_@NF and Co_0.4_NiS@NF were evaluated by linear sweep voltammetry curves (LSV, without IR compensation) in 1.0 m KOH aqueous solution with and without 50 × 10^−3^ m HMF. For OER, the potentials were swept from 1.7 V versus reversible hydrogen electrode (RHE, in following discussions, only the number of potential will be shown) to 0.8 V in order to minimize the influence of the oxidation of Ni or/and Co ions on the onset potentials and current densities (two colored dot‐dash line, **Figure** **3**a). After sulfidation, Ni_3_S_2_@NF requires an onset potential (*η*_0_) of 1.49 V to achieve a current density of 1.0 mA cm^–2^ and a potential (*η*_10_) of 1.58 V to reach 10 mA cm^–2^ (blue dot‐dash line). For Co_0.4_NiS@NF (red dot‐dash line), these two potentials decreased to 1.413 and 1.47 V, respectively. These values are comparable to those of the reported Ni/Co‐based electrocatalyst.^[^ ^48^ ^]^ In the cases with the presence of 50 × 10^−3^ m HMF, the sweepings started from 0.8 V to higher potentials (two colored solid line, Figure 3a). The LSV curves for HMFOR using Ni_3_S_2_@NF and Co_0.4_NiS@NF both shifted toward lower potentials. It is worth mentioning that the onset potential for HMFOR using Co_0.4_NiS@NF shifted to ≈0.90 V, which is lower than that of OER (about 513 mV) and those of previously reported catalysts (Table S2, Supporting Information). Since the onset potential represents the thermodynamic aspect of the electrochemical system,^[^ ^14^ ^]^ the HMFOR is thermodynamically more favorable than OER. The HMFOR potentials for reaching current densities of 50, 100, and 200 mA cm^−2^ by Co_0.4_NiS@NF were 1.24, 1.29, and 1.34 V, respectively, appreciably lower (at least 312 mV) than those for OER (1.55, 1.62, and 1.72 V) (Figure 3b).

The OER and HMFOR performances of NF, Ni_3_S_2_@NF, Co_0.1_NiS@NF, Co_0.2_NiS@NF, and Co_0.6_NiS@NF were also investigated (Figure S4, Supporting Information). The NF showed inferior performances for both OER and HMFOR. Compared with Ni_3_S_2_@NF, all the Co_x_NiS@NF catalysts exhibited enhanced electrooxidation performance as evidenced by the shifting of potentials to lower values (Figure S4a, Supporting Information). The anodic peaks located in the range of 1.2–1.4 V are believed to be originated from the in situ reconstructions of catalyst surfaces and formations of oxidation species such as M(oxy) hydroxides (M = Ni, Co), which can serve as the functional active species for OER and HMFOR.^[^ ^49^, ^50^ ^]^ The potential threshold for reconstruction (Figure S4a, Supporting Information) gradually decreased as the Co content increased from *x* = 0.1 to *x* = 0.4. However, excessive amounts of Co (0.6 mmol) in Co_0.6_NiS@NF, offset the improvement of the activity of the catalyst. The current densities of all catalysts for HMFOR increased rapidly after their reconstruction potentials (Figure S4b, Supporting Information). The OER and HMFOR performances of Co*_x_* NiS@NF catalysts involve similar trends, indicating there is an optimal doping content of Co. For all the catalysts, the current densities (Figure S4b, Supporting Information) for HMFOR are higher than those in OER processes. At potentials of 1.35, 1.4, and 1.45 V, the current densities using the Co_0.4_NiS@NF can reach 244, 368, and 497 mA cm^–2^ for HMFOR (Figure 3c), which are about 11.8, 4.5, and 4.7 times of those of Ni_3_S_2_@NF and significantly higher than those previously reported values (Table S3, Supporting Information).

The Co‐doping in the Ni_3_S_2_@NF also exerts an influence on the electrooxidation kinetics. As shown in Figure 3d, the Tafel slopes of the Ni_3_S_2_@NF and Co_0.4_NiS@NF were calculated to be 480 and 199 mV dec^–1^ for OER and 145 and 94 mV dec^–1^ for HMFOR, accordingly. For both processes, the Tafel slopes decreased as a result of Co incorporation, implying that higher electron transfer rates could be achieved via Co doping.^[^ ^12^ ^]^ Meanwhile, the Tafel slopes of the HMFOR are lower than those of OER processes using the two catalysts. This clearly manifests that the interfacial electron transfer between the HMF molecule and the catalyst could be easier, which result a lower adsorption potential of HMF to the electrode. The Nyquist plot of Co_0.4_NiS@NF (Figure 3e) exhibits a smaller diameter semicircle than that of Ni_3_S_2_@NF, further proving that a faster charge transfer could be attained on its surface.

In order to gain insight into the intrinsic activities of Co_0.4_NiS@NF and Ni_3_S_2_@NF, their electrochemical surface areas (ECSA) in HMFOR were compared by estimating their electrochemical double‐layer capacitance (*C*_dl_) in the non‐faradaic regions using cyclic voltammetry (CV) curves (Figure S5, Supporting Information).^[^ ^14^ ^]^ As shown in Figure S5 (Supporting Information) and Figure 3f, the current densities in the CV curves of Co_0.4_NiS@NF are much higher than those of Ni_3_S_2_@NF. The *C*_dl_ values of Co_0.4_NiS@NF and Ni_3_S_2_@NF are calculated to be 122.9 and 17.3 mF cm^−2^, respectively (Figure 3f). This clearly suggests that doping Co into Ni_3_S_2_@NF results in a dramatic increase of *C*_dl_ value as well as ECSA. These results, combined with its uniform dense nanosheet‐like structure, verify that more active sites could be exposed and the electron/charge transfer and/or rapid mass transfer of the HMF molecule could be facilitated on the Co_0.4_NiS@NF electrocatalyst.

### 2.3 Chronoamperometry Electrolysis Measurements

**Figure 4:**
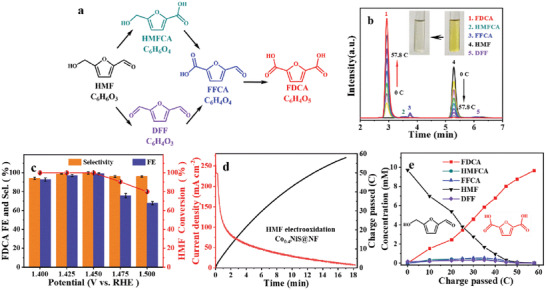
a) The possible pathways of HMF oxidation to FDCA. The chronoamperometric electrolysis measurements of HMFOR by Co_0.4_NiS@NF at different applied potentials in 10 mL 1.0 m KOH with 10 × 10^−3^ m HMF. b) HPLC chromatogram as charge accrues at 1.45 V (inset: color change of electrolyte before and after electrolysis). c) FE, selectivity of FDCA, and the HMF conversion at different potentials. d) The current and charge versus electrooxidation time and e) the concentration changes of HMF and its oxidation products during HMF electrooxidation at 1.45 V.

**Figure** **4**a shows there are two possible oxidation routes for HMF, via the oxidation of either CHO or OH group on its Furan ring to form their corresponding intermediates, e.g., 5‐hydroxymethyl‐2‐furancarboxylic acid (HMFCA) or 2,5‐diformylfuran (DFF), to be oxidized into FDCA.^[^ ^6^ ^]^ Both pathways merge at another intermediate, 2‐formyl‐5‐furancarboxylic acid (FFCA). The chronoamperometric electrolysis measurements of HMF electrooxidation on Co_0.4_NiS@NF were conducted in an H‐type electrochemical cell. HPLC was utilized to monitor and quantify the concentration changes of HMF, intermediates, and the oxidation products. Since the oxidation of one HMF molecule to one FDCA molecule needs six electrons, a theoretical charge of 57.8 C is required for completely converting 10^–4^ mole of HMF (10 × 10^−3^ m HMF in 10 mL electrolyte) into FDCA. From the HPLC chromatogram obtained at 1.45 V (Figure 4b), the peak intensity of HMF decreases gradually while the peak intensity of FDCA increases as the charge accrues from 0 to 57.8 C. Meanwhile, the color of the electrolyte is changed from the initial yellow HMF solution to almost colorless FDCA solution after electrolysis (the inset image in Figure 4b). The HMF electrooxidations were systematically conducted at the potentials of 1.4, 1.425, 1.45, 1.475, and 1.5 V. The concentration changes of all five chemicals versus the charge passing through the cell at each potential were presented in Figure S6 (Supporting Information) using the calibration curves (Figure S7, Supporting Information). Since the current density increases with increasing applied potential, the time to reach 57.8 C decreases from 61.4 to 8 min (Figure S6f, Supporting Information). The calculated conversions of HMF, selectivity, and FEs of FDCA were plotted in Figure 4c. Higher conversions of HMF and FEs of FDCA can be achieved at lower potentials (1.4, 1.425, and 1.45 V). As the potential rises further (1.475, 1.5 V), the conversion of HMF and FE of FDCA gradually decrease due to the occurrence of the competitive OER. Although both oxidation pathways are involved in the HMFOR using Co_0.4_NiS@NF catalyst, the selectivity of FDCA is great than 94% at all potentials. The FE of FDCA increases from 92.6% at 1.4 V to 99.1% at 1.45 V and then decreases to 67.6% at 1.5 V. At 1.4 and 1.425 V, it takes a longer time for the HMFOR because the current density is rather small. Therefore, the relatively low FEs at these two potentials might be attributed to the degradation of HMF as a result of the slow oxidation of HMF into the intermediates. The FDCA yield rate increased from 90.5 to 506.7 µmol cm^–2^ h^–1^ along with the increase of applied potential (Table S4, Supporting Information), also due to the enhanced reaction kinetics. These results clearly reveal that the optimal potential for HMF electrooxidation to FDCA is 1.45 V.

For HMF oxidation into FDCA using Co_0.4_NiS@NF as the electrocatalyst at 1.45 V, along with the increase of charge, the current density gradually decreases as a result of progressive consumption of HMF (Figure 4d). The concentration changes of HMF, three major intermediates, and FDCA during the oxidation reaction were depicted in Figure 4e. It is worth mentioning that the concentrations of HMFCA, DFF, and FFCA are all below 1.0 × 10^−3^ m during the whole electrooxidation process without accumulation, which further confirms that an excellent reaction kinetics for HMFOR could be attained on the Co_0.4_NiS@NF electrocatalyst. Since it only takes 18 ± 2 min to reach the theoretical charge of 57.8 C, such a rapid conversion of HMF can effectively prevent the degradation of HMF under strong alkaline conditions. The attained nearly 100% conversion of HMF and high selectivity and FE of FDCA (both higher than 99%) make our optimized Co_0.4_NiS@NF catalyst to be ranked among the top of the reported electrocatalysts for HMFOR (Table S2, Supporting Information).

### 2.4 Stability of Co_0.4_NiS@NF for HMFOR

**Figure 5:**
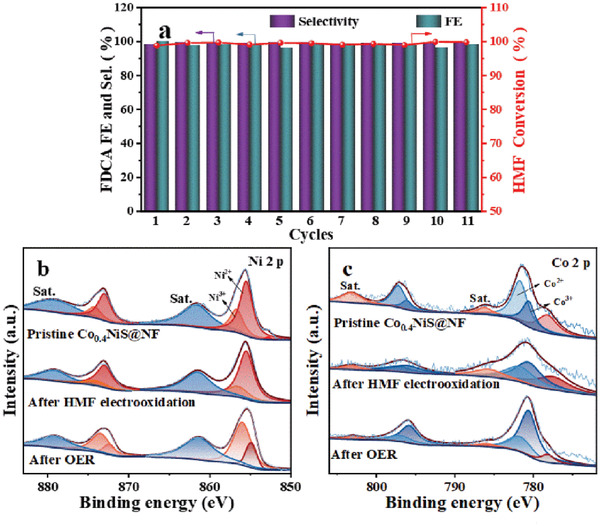
a)The FE and selectivity of FDCA, and the conversion of HMF obtained by the Co_0.4_NiS@NF for eleven consecutive cycles of HMFOR. High‐resolution XPS spectra of b) Ni 2p, c) Co 2p of Co_0.4_NiS@NF before and after HMFOR and OER.

The cycling stability of the Co_0.4_NiS@NF electrode for HMFOR was carried out by applying the constant potential of 1.45 V in a 10 mL electrolyte containing 1.0 m KOH and 10 × 10^−3^ m HMF. After passing a total charge of 57.8 C for each run, the electrolyte was refreshed by the same 10 mL electrolyte. The current density over eleven successive cycles remains almost unchanged (Figure S8, Supporting Information). The HMF conversion (98.8%–100%) as well as the selectivity (98.5%–99.9%) and EF of FDCA (96.3%–99.9%) fluctuate in narrow ranges (**Figure** **5**a), demonstrating the excellent stability of Co_0.4_NiS@NF for HMFOR. Each cycle can be finished within 18 ± 4 min for reaching the theoretical charge (57.8 C) to convert HMF into FDCA. XRD pattern of the Co_0.4_NiS@NF after eleven consecutive cycles shows that Ni_3_S_2_ and NF are still the two main crystalline phases (Figure S9, Supporting Information).

XPS spectra of pristine Co_0.4_NiS@NF and the catalysts after OER and HMFOR are shown in Figure 5b,c. Both OER and HMFOR were carried out under 1.45 V in 10 mL 1.0 m KOH electrolyte with and without 10 × 10^−3^ m HMF. Compared with pristine Co_0.4_NiS@NF, the ratio of Ni^3+^/Ni^2+^ decreases from 2.3 to 0.3 after HMFOR, and increases to 4.1 after OER. Meanwhile, the ratios of Co^3+^/Co^2+^ slightly increased from 0.3 to 1.3 after HMFOR and to 2.4 after OER (Table S5, Supporting Information). Since the ratios of Ni^3+^/Ni^2+^ and Co^3+^/Co^2+^ are all increased after OER, both Ni^2+^ and Co^2+^ species are partially oxidized to high valence Ni^3+^ and Co^3+^. This is consistent with the reconstruction of the catalyst's surface to form the functional oxidation state of NiCo (oxy)hydroxides.^[^ ^49^ ^]^ After HMFOR, the decrease of Ni^3+^/Ni^2+^ and a slight increase of Co^3+^/Co^2+^ ratios suggest that the phase reconstruction might not be favored during HMFOR.

**Figure 6:**
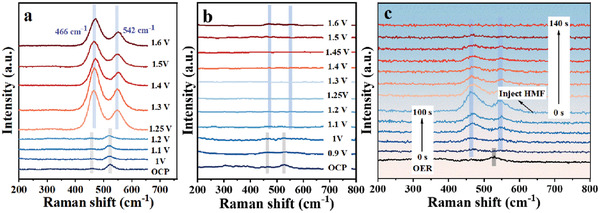
In situ Raman spectra of Co_0.4_NiS@NF electrodes during a) OER (1.0 m KOH) and b) HMFOR (1.0 m KOH with 50 × 10^−3^ m HMF) under increasing potential from OCP to 1.6 V and c) OER (1.0 m KOH) and half‐way injected HMF (10 × 10^−3^ m HMF) under 1.45 V.

To further investigate the HMFOR mechanism on Co_0.4_NiS@NF catalyst, in situ Raman spectra were conducted to monitor the surface structure during OER and HMFOR at different potentials (**Figure** **6**a,b). Each Raman spectrum shown in these two cases was obtained after collecting the data after 120 s running. At the open circuit potential (OCP), there are typical Raman peaks at 460 and 531 cm^–1^ for Co_0.4_NiS@NF.^[^ ^4^, ^51^ ^]^ Increasing the applied potential of OER to 1.25 V, new characteristic Raman peaks of the NiCo (oxy)hydroxides at 466 and 542 cm^–1^ were detected. This is in good agreement with the formation of anodic peaks located in the range of 1.2–1.3 V in the LSV curve shown in Figure S4 (Supporting Information) and further confirm the reconstruction of the catalyst (Figure 6a).^[^ ^9^, ^52^ ^]^ For HMFOR, such NiCo(oxy)hydroxides species could be found only at 1.60 V or above (Figure 6b). Figure S10 (Supporting Information) shows the Raman test for the organic substrates (HMF, FDCA, DFF, FFCA, and HMFCA) and no significant peaks appear. To unveil the cause of such inconsistency, an electrolysis process was first run without the presence of HMF (the OER) for 100 s and then with the presence of 10 × 10^−3^ m HMF (the HMFOR) for 140 s at 1.45 V (Figure 6c). At the transition point, HMF was injected into the cell to induce the HMFOR. During the OER process in the first 100 s electrolysis, the characteristic peaks associated with Co_0.4_NiS@NF quickly disappear along with the emerging and progressive growth of NiCo (oxy)hydroxides peaks. Once HMF was injected in a halfway manner, the peaks of NiCo (oxy)hydroxides faded away within 140 s. It has been widely accepted that the generated high valence states (NiCo (oxy)hydroxides) are the real catalytic sites for both OER and HMFOR.^[^ ^8^, ^53^ ^]^ Comparison between Figure 6a,b clearly reveals that HMFOR is more favorable than OER under these electrolytic conditions. As nearly no high valence states (NiCo (oxy)hydroxides) could be detected in Figure 6b, it is reasonable to deduce that the generation of NiCo (oxy)hydroxides is the rate‐determining step and the HMFOR occurs quite fast.^[^ ^9^, ^54^ ^]^ Combined with electrochemical measurement results, at the applied potential of 1.45 V, the rates in forming high valence active species and converting HMF into its oxidized intermediates or final FDCA nearly reach a balanced state. Lower potential means the production of high valence active species is low, resulting in a slow HMFOR. While OER emerged as a competitive reaction when the potential higher than 1.45 V, resulting in a negative effect on the efficiency of HMFOR. In the meantime, the formation rate of high valence active species increased makes it detectable in the Raman spectroscopy.

### 2.5 Effects of Reaction Temperature and HMF Concentration

The effects of electrolysis temperature and HMF concentration on HMFOR in the same electrolyte using the Co_0.4_NiS@NF electrocatalyst were also investigated. When the temperature is set at 25, 35, 45, and 55 °C (Figure S11, Supporting Information), the electrolysis time required to reach the theoretical 57.8 C gradually decreases, indicative of an increase of reaction rate along with elevating reaction temperature. The concentrations of the five chemicals including HMF, DFF, HMFCA, FFCA, and FDCA versus charge passed and the conversion of HMF, selectivity, and FE of FDCA at each temperature were summarized in Figures S12 and S13 (Supporting Information) (also using the calibration curves in Figure S7, Supporting Information). Since the self‐degradation of HMF under alkaline conditions through the Cannizzaro reaction in forming the Humin‐type products could be also expedited,^[^ ^26^, ^55^ ^]^ the FE of FDCA decreases from 99.1% to 80.6% as the temperature increases (Figure S13, Supporting Information). Accompanied with the decrease of FE, the self‐degradation of HMF results in carbon imbalances (Figure S14, Supporting Information). However the high temperature does favor the HMFOR as the high yield rate of FDCA could be obtained at elevated temperatures (Table S6, Supporting Information).

In terms of the reactant concentration, the electrooxidation of HMF with various concentrations was studied at 5 × 10^−3^, 10 × 10^−3^, 20 × 10^−3^, 30 × 10^−3^, 50 × 10^−3^, and 100 × 10^−3^ m at 25 °C. With the increase in HMF concentration, the time required for the electrolysis is prolonged (Figure S15, Supporting Information) and the color of the electrolyte becomes darker due to the formation of black Humin with a degradation rate of ≈10% per hour (Figure S16, Supporting Information). The concentration changes of HMF, DFF, HMFCA, FFCA, and FDCA versus charge applied using different amounts of HMF are shown in Figure S17 (Supporting Information). Again, the conversion of HMF, selectivity, and FE of FDCA at different temperatures are calculated and plotted in Figure S18 (Supporting Information). Combining the electrolysis time and FE of FDCA, the yield rates of FDCA using different concentrations of HMF are shown in Table S7 (Supporting Information). Specifically, when the concentration of HMF is 20 × 10^−3^ m, the yield rate of FDCA is calculated to be 488.3 µmol cm^–2^ h^–1^ after 24.3 min electrolysis time, with a conversion of HMF close to 100% and 100% selectivity of FDCA. Further increasing HMF concentration, the FE of FDCA decreases gradually as a result of HMF degradation paralleled with the carbon imbalance (Figure S19, Supporting Information), although the conversion of HMF and the selectivity of FDCA remained nearly unchanged at high levels. This phenomenon illustrates the necessity of developing catalysts with large current densities to achieve rapid electrolytic HMF oxidation. The combined test results show that Co_0.4_NiS@NF is suitable as a catalyst for HMFOR in the concentration range of 10–20 × 10^−3^ m HMF.

### 2.6 Coupling HER and HMFOR

Above results unambiguously demonstrated that our Co_0.4_NiS@NF is an efficient electrocatalyst for HMFOR. For the coupling of HMFOR with HER, the Co_0.4_NiS@NF was used as the anode electrocatalyst while the Pt electrode was used as the cathode as shown in Figure S20 (Supporting Information). The productions of H_2_ and FDCA were quantified after passing a total charge of 57.8 C in 10 mL electrolyte containing 1.0 m KOH and 10 × 10^−3^ m HMF. For comparison, pure water splitting was also tested in the absence of HMF. The HMF conversion, selectivity, and FE of FDCA are comparable with those obtained in 11 cycles of stability tests. The yield rate of FDCA is about 330.4 µmol cm^–2^ h^–1^. For the cathode chamber, a nearly 100% FE, given from gas chromatography (GC) analysis, could be achieved. This corresponds to an extremely high H_2_ production rate of 1000 µmol cm^–2^ h^–1^. In contrast, the H_2_ production rate for HER paired with OER is only 97.8 µmol cm^–2^ h^–1^ (Figure S21, Supporting Information). Clearly, replacing OER with the HMFOR can effectively promote the H_2_ production, reduce energy consumption, and enhance atom utilization.

The electrooxidation performances of other four biomass‐derived platform substrates (glucose, fructose, furfural, and benzoic acid) were also evaluated under similar conditions. The performances toward the electrooxidation of these four substrates using Co_0.4_NiS@NF were also evaluated by LSV in 1.0 m KOH aqueous solution with and without 50 × 10^−3^ m of each substrate (Figure S22, Supporting Information). As can be seen from Figure S22, Supporting Information, the onset potentials for all four substrates shifted to approximately 0.9 V. Their current densities rapidly rise at potential beyond 1.2 V. The integrated electrolysis is a promising method for the production of H_2_ and value‐added chemicals derived from biomass products at an industrial scale. Due to the complex structure of most biomass‐derived platform substrates and reaction intermediates, their reaction mechanisms and theoretical studies should be further explored.

## 3 Conclusion

The Co‐doped Ni_3_S_2_@NF as an efficient and low‐cost electrocatalyst for HMF oxidation was successfully synthesized through a simple hydrothermal method. The XRD and XPS results confirmed that Co was successfully incorporated into the lattice of Ni_3_S_2_ in Co‐doped Ni_3_S_2_@NF, leading to lattice distortions and a decrease in crystallinity. The optimized Co_0.4_NiS@NF exhibited excellent HMFOR activity with an ultralow onset potential (0.9 V vs RHE) and a high current density (497 mA cm^–2^) at the applied potential of 1.45 V versus RHE. When the electrolysis was carried out in 10 mL electrolyte containing 1.0 m KOH and 10 × 10^−3^ m HMF, it can be fully converted into FDCA within 18 ± 2 min. The Co_0.4_NiS@NF electrocatalyst displayed a good cycling durability toward HMFOR. To minimize the self‐degradation of HMF, the electrooxidation of HMF should be performed under lower temperature. For the HMFOR‐assisted H_2_ production, the yield rates of FDCA and H_2_ are about 330 and 1000 µmol cm^–2^ h^–1^, respectively, at 1.45 V versus RHE. This performance is superior to most of the reported electrocatalysts and meets the requirements for industrial large‐scale production of FDCA and H_2_. The excellent performance for HMF electrooxidation could be attributed to its highly porous nature of Co_0.4_NiS@NF and Co doping into Ni_3_S_2_@NF. Such doping results in a dramatic increase of ECSA, which allowed the expoure of more active sites and effectively modulates the electronic properties of Ni_3_S_2_. These facilitated the electron/charge transfer and/or rapid mass transfer processes and in turn promoted the catalytic activity of the sample. In situ Raman studies reveal that the surface of Co_0.4_NiS@NF was reconstructed quickly into NiCo (oxy) hydroxides, which reduced the potential threshold and acted as the actual active sites for the OER and HMFOR. The extension of using Co_0.4_NiS@NF as catalyst for the electrooxidations of other bio‐mass derived chemicals was proven to be feasible. This finding of this study pave a way for the development of industrial‐level H_2_ and FDCA production methodologies based on the couping of HMFOR and HER.

## 4 Experimental Section

### Chemicals and Materials

5‐Hydroxymethylfurfural (HMF, 95%), 2,5‐furandicarboxylic acid (FDCA, 98%), 2‐formyl‐5‐furancarboxylic acid (FFCA, 98%), 2,5‐diformyfuran (DFF, 98%), 5‐hydroxymethyl‐2‐furancarboxylic acid (HMFCA, 98%), potassium hydroxide (KOH), cobaltous chloride (CoCl_2_·6H_2_O), methanol, and ammonium formate were obtained from Sigma‐Aldrich. Nickel foam (NF, thickness 1.6 mm) was purchased from Suzhou Shuertai Industrial Technology Co., Ltd., China. Methanol is of chromatographic pure grade, and the other chemicals are of analytical grade.

### Preparation of Co‐Doped Ni_3_S_2_ Supported on Nickel Foam (Co_0.1_NiS@NF, Co_0.2_NiS@NF, Co_0.4_NiS@NF, and Co_0.6_NiS@NF) and Ni_3_S_2_@NF

Nickel foam (NF) was cut into small pieces with dimensions of 2.0 × 3.0 cm^2^. These small pieces were ultrasonicated for 10 min with an aqueous solution (3.0 m HCl), ethanol, and ultrapure water, respectively.^[^ ^56^ ^]^ Taking Co_0.4_NiS@NF as an example, a mixed solution of CoCl_2_·6H_2_O (0.4 mmol) and thiourea (1.5 mmol) dissolved in ultrapure water (30 mL) was prepared and transferred into a stainless steel autoclave with Teflon liner (50 mL). Then a piece of small NF was immersed vertically into the autoclave reactor. Subsequently, the autoclave was heated in a preheated oven at 140 °C for 12 h. After reaction, the autoclave reactor was cooled down to room temperature naturally. The prepared Co_0.4_NiS@NF was taken out and rinsed thoroughly with ultrapure water. Thereafter, it was dried inside a vacuum oven at 60 °C for 12 h. The preparations of Ni_3_S_2_@NF, Co_0.1_NiS@NF, Co_0.2_NiS@NF, and Co_0.6_NiS@NF were similar to Co_0.4_NiS@NF except for varying the amounts of Co precursor (CoCl_2_·6H_2_O) from 0 to 0.1, 0.2, and 0.6 mmol.

### Material Characterizations

The crystallinity and phases of the samples were characterized by X‐ray diffraction (XRD, SmartLab 9KW, Rigaku, Japan). The morphologies of the prepared materials were observed by a scanning electron microscope (SEM, Verios 460L, USA) and transmission electron microscopy (TEM, TECNAL G2 Spirit TWIN) equipped with a LaB6 emission gun. The chemical composition and valence state of the elements were determined by X‐ray photoelectron spectroscopy (XPS, ESCALAB250Xi, Thermo Scientific, UK). All the binding energies were calibrated relative to C 1s (284.8 eV). The Ni and Co contents of as‐prepared catalysts were quantified by inductivity coupled plasma‐atomic emission spectrometry (ICP‐AES, Thermo Scientific).

### Electrochemical Measurements

Except for constant potential electrolysis measurement, all other electrochemical measurements were performed with a Metrohm electrochemical workstation in an H‐type cell (10 mL), in which the two chambers were separated by a Nafion 117 proton exchange membrane. The constant potential electrolysis measurements were performed with a CHI‐660E electrochemical analyzer. The electrochemical tests were carried out with the three‐electrode configuration, the as‐synthesized catalyst on Ni foam (≈1.0 × 1.0 cm) was directly used as the working electrode, a mercury/mercury oxide (Hg/HgO) and a graphite rod were used as the reference electrode and counter electrode, respectively. During the paired electrolysis, the counter electrode was a platinum sheet (1.0 × 1.0 cm). 1.0 m KOH (pH 13.8) with or without different concentrations of HMF were used as the electrolytes. Linear sweep voltammetry (LSV) was collected until the test results kept stable at a scan rate of 5 mV s^−1^. All potentials in this study were referred to the reversible hydrogen electrode (RHE) according to the following equation without IR compensation:

(1) $${E_{\mathrm{vs}}}_{\mathrm{RHE}}={E_{\mathrm{vs}.}}_{\mathrm{Hg}/\mathrm{HgO}}+0.098\;V+0.0592\mathrm{pH}$$

### Estimation of the Effective Electrode Surface Area

The electrochemical surface area (ECSA) of the electrocatalyst was estimated from the electrochemical double‐layer capacitance (*C*_dl_), which was investigated via cyclic voltammetry (CV) cycles. The CV was performed in 1.0 m KOH containing 50 × 10^−3^ m HMF at various scan rates of 1, 3, 5, 7, 9, and 10 mV s^–1^ in a non‐faradaic potential window.

### Measurements of the Electrochemical Impedance Spectroscopy (EIS)

Electrochemical impedance spectroscopy (EIS) tests were measured over a frequency range from 10^–1^ to 10^5^ Hz with an AC amplitude of 10 mV. The tests were carried out with the typical three‐electrode configuration at the open circle voltage.

### In Situ Raman Spectroscopy

In situ Raman spectra were carried out on a laser confocal Raman spectroscopy (HORIBA EVOLUTION, France). The electrolytic cell for in situ Raman Spectrum comprises a Teflon shell, quartz glass plate, Pt wire, and Ag/AgCl electrodes. All the electrochemical tests were carried out using the three‐electrode configuration connected to an electrochemical workstation (CHI‐660E). The counter electrode was a platinum wire for HER. The electrolyte is 1.0 m KOH with or without HMF for the HMF electrooxidation and OER testing.

### Quantitative Analysis of Products

The HMF, intermediates (HMFCA, FFCA, HMF, and DFF) and oxidation product (FDCA) were analyzed by high‐performance liquid chromatography (HPLC, Shimadzu Prominence LC 2030 Plus system) with an ultraviolet (UV)–visible detector and a Shim‐pack GWS C18 (5 µm, 4.6 ×150 mm) column. In a typical experiment, 20 µL of electrolyte was sampled during the electrolysis, diluted to 1 mL with ultrapure water, and analyzed by HPLC. As for the analysis conditions, the wavelength of the UV detector was 265 nm, the mobile phase A was methanol, and phase B was 5 × 10^−3^ m ammonium formate aqueous solution. The volume ratio of A/B is 3:7 and the flow rate is 0.5 mL min^–1^. The column temperature was maintained at 40 °C, and each separation lasts for 8 min.

The conversion of HMF and the selectivity, faradaic efficiency (FE), and yield rate of FDCA were calculated according to Equations (2–5), respectively.

(2) $$\mathrm{HMF}\;\mathrm{conversion}\;(\%)\;=\frac{\text{mol}\;\text{of}\;\text{HMF}\;\text{consumed}}{\text{mol}\;\text{of}\;\text{HMF}\;\text{initial}}\;\;\times 100\%$$

(3) $$\text{FDCA}\;\text{selectivity}\;\;\left(\%\right)=\frac{\text{mol}\;\text{of}\;\text{FDCA}\;\text{formed}}{\text{mol}\;\text{of}\;\text{HMF}\;\text{consumed}}\;\;\times 100\%$$

(4) $$\text{FE}\;\;\left(\%\right)=\frac{\text{mol}\;\text{of}\;\text{FDCA}\;\text{formed}\;}{\;(\mathrm{charge}\;/\;(6\;\times F))}\;\;\times 100\%$$

(5) $$\mathrm{FDCA}\;\mathrm{yield}\;\mathrm{rate}=\frac{\mathrm{mol}\;\mathrm{of}\;\mathrm{FDCA}\times 1000\;}{S\times t}$$

The mol of HMF and FDCA is measured by HPLC. With the *F* represents the Faraday constant (96 485 C mol^–1^), the *S* is the electrode geometric area, and *t* is the electrolysis time (*h*).

The H_2_ test was carried out in an H‐type cell (air‐tight), in which the cathode gas sample was pumped into the gas chromatograph (GC, Agilent 7890 B). The electrolyte was purged with argon (Ar) for 0.5 h prior to measurements. Pure Ar was also used as the carrier gas. The gas sample (1 mL) was pumped into the GC at the headspace position of the cathode via online sampling.

## Conflict of Interest

The authors declare no conflict of interest.

## Supporting information

Supporting Information

## Data Availability

The data that support the findings of this study are available in the supplementary material of this article.

## References

[1] Z. Y. Yu , Y. Duan , X. Y. Feng , X. Yu , M. R. Gao , S. H. Yu , Adv. Mater. 2021, 33, 2007100. 10.1002/adma.202007100 34117808

[2] B. Rausch , M. D. Symes , G. Chisholm , L. Cronin , Science 2014, 345, 1326. 25214625 10.1126/science.1257443

[3] X. Zou , Y. Liu , G. D. Li , Y. Wu , D. P. Liu , W. Li , H. W. Li , D. Wang , Y. Zhang , X. Zou , Adv. Mater. 2017, 29, 1700404.

[4] M. F. Lagadec , A. Grimaud , Nat. Mater. 2020, 19, 1140. 33020614 10.1038/s41563-020-0788-3

[5] X. Liu , R. Guo , K. Ni , F. Xia , C. Niu , B. Wen , J. Meng , P. Wu , J. Wu , X. Wu , L. Mai , Adv. Mater. 2020, 32, 2001136. 10.1002/adma.202001136 32876959

[6] B. You , X. Liu , N. Jiang , Y. Sun , J. Am. Chem. Soc. 2016, 138, 13639. 27652996 10.1021/jacs.6b07127

[7] C. Cao , D. D. Ma , J. Jia , Q. Xu , X. T. Wu , Q. L. Zhu , Adv. Mater. 2021, 33, 2008631. 10.1002/adma.202008631 33988264

[8] H. B. Tao , Y. H. Xu , X. Huang , J. Z. Chen , L. J. Pei , J. M. Zhang , J. G. G. Chen , B. Liu , Joule 2019, 3, 1498.

[9] H. Huang , C. Yu , X. Han , H. Huang , Q. Wei , W. Guo , Z. Wang , J. Qiu , Energy Environ. Sci. 2020, 13, 4990.

[10] R. Mariscal , P. Maireles‐Torres , M. Ojeda , I. Sádaba , M. L. Granados , Energy Environ. Sci. 2016, 9, 1144.

[11] X. Liu , B. Li , G. Han , X. Liu , Z. Cao , D. E. Jiang , Y. Sun , Nat. Commun. 2021, 12, 1868. 33767166 10.1038/s41467-021-22157-5 PMC7994825

[12] W. J. Liu , Z. Xu , D. Zhao , X. Q. Pan , H. C. Li , X. Hu , Z. Y. Fan , W. K. Wang , G. H. Zhao , S. Jin , G. W. Huber , H. Q. Yu , Nat. Commun. 2020, 11, 265. 31937783 10.1038/s41467-019-14157-3 PMC6959317

[13] W. Guan , Y. L. Zhang , Y. N. Wei , B. Li , Y. H. Feng , C. H. Yan , P. W. Huo , Y. S. Yan , Fuel 2020, 278, 118362.

[14] Y. Chen , B. Tian , Z. Cheng , X. Li , M. Huang , Y. Sun , S. Liu , X. Cheng , S. Li , M. Ding , Angew. Chem., Int. Ed. 2021, 60, 4199. 10.1002/anie.202014072 33180375

[15] T. Wu , K. D. Moeller , Angew. Chem., Int. Ed. 2021, 60, 12883. 10.1002/anie.202100193 33768678

[16] C. Xu , E. Paone , D. Rodriguez‐Padron , R. Luque , F. Mauriello , Chem. Soc. Rev. 2020, 49, 4273. 32453311 10.1039/d0cs00041h

[17] X. Huang , J. L. Song , M. L. Hua , Z. B. Xie , S. S. Liu , T. B. Wu , G. Y. Yang , B. X. Han , Green Chem. 2020, 22, 843.

[18] Y. Xie , Z. Zhou , N. Yang , G. Zhao , Adv. Funct. Mater. 2021, 31, 2102886.

[19] A. J. J. E. Eerhart , A. P. C. Faaij , M. K. Patel , Energy Environ. Sci. 2012, 5, 6407.

[20] T. Werpy , G. Petersen , Top Value Added Chemicals from Biomass: Volume I – Results of Screening for Potential Candidates from Sugars and Synthesis Gas, United States, N. p., 2004. https://www.osti.gov/biblio/15008859 (accessed: 2004).

[21] M. Y. Chen , C. B. Chen , B. Zada , Y. Fu , Green Chem. 2016, 18, 3858.

[22] B. A. Frontana‐Uribe , R. D. Little , J. G. Ibanez , A. Palma , R. Vasquez‐Medrano , Green Chem. 2010, 12, 2099.

[23] Y. Y. Zhao , M. K. Cai , J. H. Xian , Y. M. Sun , G. Q. Li , J. Mater. Chem. A 2021, 9, 20164.

[24] W. Liu , Y. Cui , X. Du , Z. Zhang , Z. S. Chao , Y. L. Deng , Energy Environ. Sci. 2016, 9, 467.

[25] Y. C. Yang , T. C. Mu , Green Chem. 2021, 23, 4228.

[26] S. Barwe , J. Weidner , S. Cychy , D. M. Morales , S. Dieckhofer , D. Hiltrop , J. Masa , M. Muhler , W. Schuhmann , Angew. Chem., Int. Ed. 2018, 57, 11460. 10.1002/anie.201806298 29985550

[27] Y. Lu , C. L. Dong , Y. C. Huang , Y. Zou , Z. Liu , Y. Liu , Y. Li , N. He , J. Shi , S. Wang , Angew. Chem., Int. Ed. 2020, 59, 19215. 10.1002/anie.202007767 32705755

[28] Y. Lu , T. Liu , C. L. Dong , Y. C. Huang , Y. Li , J. Chen , Y. Zou , S. Wang , Adv. Mater. 2021, 33, 2007056. 10.1002/adma.202007056 33470476

[29] F. J. Holzhäuser , T. Janke , F. Öztas , C. Broicher , R. Palkovits , Adv. Sustainable Syst. 2020, 4, 1900151.

[30] J. Zhang , W. Gong , H. Yin , D. Wang , Y. Zhang , H. Zhang , G. Wang , H. Zhao , ChemSusChem 2021, 14, 2935. 34013575 10.1002/cssc.202100811

[31] Q. Zhang , W. Chen , G. L. Chen , J. Huang , C. S. Song , S. J. Chu , R. Zhang , G. X. Wang , C. R. Li , K. K. Ostrikov , Appl. Catal., B 2020, 261, 118254.

[32] S. Qu , W. Chen , J. S. Yu , G. L. Chen , R. Zhang , S. J. Chu , J. Huang , X. Q. Wang , C. R. Li , K. K. Ostrikov , J. Power Sources 2018, 390, 224.

[33] X. Luo , P. X. Ji , P. Y. Wang , R. L. Cheng , D. Chen , C. Lin , J. N. Zhang , J. W. He , Z. H. Shi , N. Li , S. Q. Xiao , S. C. Mu , Adv. Energy Mater. 2020, 10, 1903891.

[34] S. Q. Qu , J. Huang , J. S. Yu , G. L. Chen , W. Hu , M. M. Yin , R. Zhang , S. J. Chu , C. R. Li , ACS Appl. Mater. Interfaces 2017, 9, 29660. 28792729 10.1021/acsami.7b06377

[35] J. Ren , M. Shen , Z. L. Li , C. M. Yang , Y. Liang , H. E. Wang , J. H. Li , N. Li , D. Qian , J. Power Sources 2021, 501, 230003.

[36] S. Ma , J. Huang , C. Zhang , G. L. Chen , W. Chen , T. Shao , T. T. Li , X. H. Zhang , T. Gong , K. K. Ostrikov , Chem. Eng. J. 2022, 435, 134859.

[37] L. Tie , N. Li , C. F. Yu , Y. M. Liu , S. Y. Yang , H. Chen , S. Y. Dong , J. Y. Sun , S. Y. Dou , J. H. Sun , ACS Appl. Energy Mater. 2019, 2, 6931.

[38] B. Fei , Z. L. Chen , J. X. Liu , H. B. Xu , X. X. Yan , H. L. Qing , M. Chen , R. B. Wu , Adv. Energy Mater. 2020, 10, 2001963.

[39] C. Jin , P. Zhai , Y. Wei , Q. Chen , X. Wang , W. Yang , J. Xiao , Q. He , Q. Liu , Y. Gong , Small 2021, 17, 2102097. 10.1002/smll.202102097 34228390

[40] L. Zhang , X. Gao , Y. Zhu , A. Liu , H. Dong , D. Wu , Z. Han , W. Wang , Y. Fang , J. Zhang , Z. Kou , B. Qian , T. T. Wang , Nanoscale 2021, 13, 2456. 33470251 10.1039/d0nr07275c

[41] B. J. Taitt , D.‐H. Nam , K.‐S. Choi , ACS Catal. 2018, 9, 660.

[42] J. Abed , S. Ahmadi , L. Laverdure , A. Abdellah , C. P. O'Brien , K. Cole , P. Sobrinho , D. Sinton , D. Higgins , N. J. Mosey , S. J. Thorpe , E. H. Sargent , Adv. Mater. 2021, 33, 2103812. 10.1002/adma.202103812 34541731

[43] C. Z. Wang , M. Z. Zhu , Z. Y. Cao , P. Zhu , Y. Q. Cao , X. Y. Xu , C. X. Xu , Z. Y. Yin , Appl. Catal., B 2021, 291, 120071.

[44] W. B. Fu , Y. Y. Zhao , J. F. Mei , F. J. Wang , W. H. Han , F. C. Wang , E. Q. Xie , Electrochim. Acta 2018, 283, 737.

[45] B. Zhou , Y. Li , Y. Zou , W. Chen , W. Zhou , M. Song , Y. Wu , Y. Lu , J. Liu , Y. Wang , S. Wang , Angew. Chem., Int. Ed. 2021, 60, 22908. 10.1002/anie.202109211 34405508

[46] S. Deng , Y. Zhong , Y. Zeng , Y. Wang , X. Wang , X. Lu , X. Xia , J. Tu , Adv. Sci. 2018, 5, 1700772. 10.1002/advs.201700772 PMC5867071 29593976

[47] Y. Chang , Y. W. Sui , J. Q. Qi , L. Y. Jiang , Y. Z. He , F. X. Wei , Q. K. Meng , Y. X. Jin , Electrochim. Acta 2017, 226, 69.

[48] Z. S. Li , B. L. Li , J. M. Chen , Q. Pang , P. K. Shen , Int. J. Hydrogen Energy 2019, 44, 16120.

[49] S. W. Boettcher , Nat. Catal. 2018, 1, 814.

[50] Y. Sun , J. Wu , Z. Zhang , Q. Liao , S. Zhang , X. Wang , Y. Xie , K. Ma , Z. Kang , Y. Zhang , Energy Environ. Sci. 2022, 15, 633.

[51] Y. Wu , Y. Liu , G.‐D. Li , X. Zou , X. Lian , D. Wang , L. Sun , T. Asefa , X. Zou , Nano Energy 2017, 35, 161.

[52] W. Chen , C. Xie , Y. Y. Wang , Y. Q. Zou , C. L. Dong , Y. C. Huang , Z. H. Xiao , Z. X. Wei , S. Q. Du , C. Chen , B. Zhou , J. M. Ma , S. Y. Wang , Chem 2020, 6, 2974.

[53] M. T. Bender , Y. C. Lam , S. Hammes‐Schiffer , K. S. Choi , J. Am. Chem. Soc. 2020, 142, 21538. 33320654 10.1021/jacs.0c10924

[54] L. Bai , S. Lee , X. Hu , Angew. Chem., Int. Ed. 2021, 60, 3095. 10.1002/anie.202011388 33089600

[55] A. Al Ghatta , X. Zhou , G. Casarano , J. D. E. T. Wilton‐Ely , J. P. Hallett , ACS Sustain. Chem. Eng. 2021, 9, 2212.

[56] X. Xiong , D. Ding , D. Chen , G. Waller , Y. Bu , Z. Wang , M. Liu , Nano Energy 2015, 11, 154.

