# Supplementary material for: Efficient Electrooxidation of 5‐Hydroxymethylfurfural Using Co‐Doped Ni3S2 Catalyst: Promising for H2 Production under Industrial‐Level Current Density
Source: Adv Sci (Weinh). 2022 Apr 15;9(17):2200957. doi: 10.1002/advs.202200957 (PMC9189636; doi:10.1002/advs.202200957)
Supplement: Supplementary file 1 — Supporting Information [file ADVS-9-2200957-s001.pdf]

ADVANCED SCIENCE
Open Access

Supporting Information

for *Adv. Sci.*, DOI 10.1002/advs.202200957

Efficient Electrooxidation of 5-Hydroxymethylfurfural Using Co-Doped Ni_3S_2 Catalyst: Promising for H_2 Production under Industrial-Level Current Density

*Yan Sun, Jie Wang, Yufeng Qi, Wenjiang Li and Cheng Wang**

Supporting Information

Efficient Electrooxidation of 5-Hydroxymethylfurfural Using Co-Doped Ni_3S_2 Catalyst: Promising for H_2 Production under Industrial-Level Current Density

*Yan Sun, Jie Wang, Yufeng Qi, Wenjiang Li and Cheng Wang**

Y. Sun, J. Wang, Y. F. Qi, Prof. C. Wang
Tianjin Key Laboratory of Advanced Functional Porous Materials, Institute for New Energy Materials & Low-Carbon Technologies, School of Materials Science and Engineering, Tianjin University of Technology, Tianjin 300384, China
E-mail: cwang@tjut.edu.cn (Prof. C. Wang).
Prof. W. J. Li

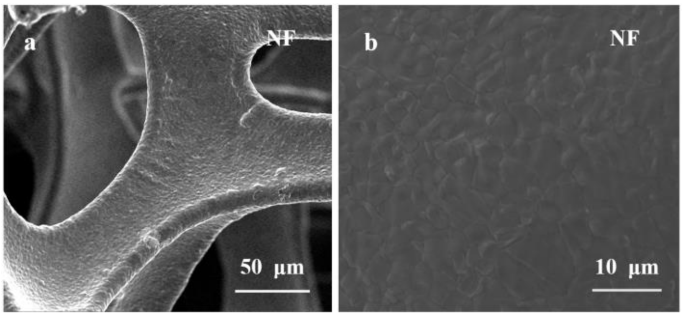

Figure S1. a) Low and b) high magnification SEM images of NF.

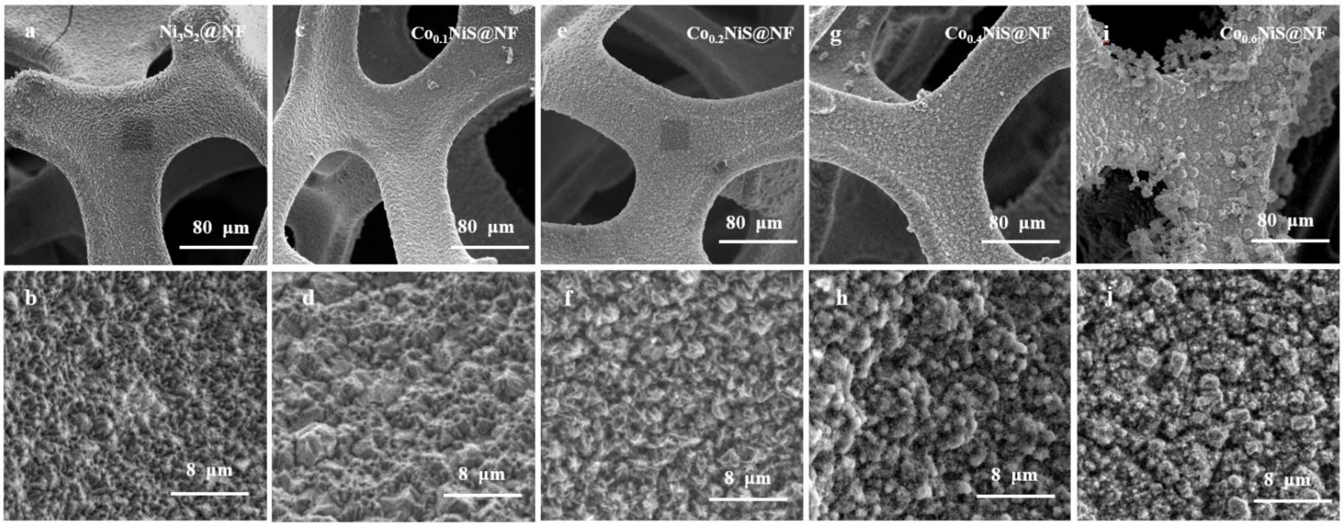

Figure S2. SEM images at low and high magnifications of a), b) Ni_3S_2@NF, c), d) $Co_{0.1}$NiS@NF, e), f) $Co_{0.2}$NiS@NF, g), h) $Co_{0.4}$NiS@NF, i), j) $Co_{0.6}$NiS@NF.

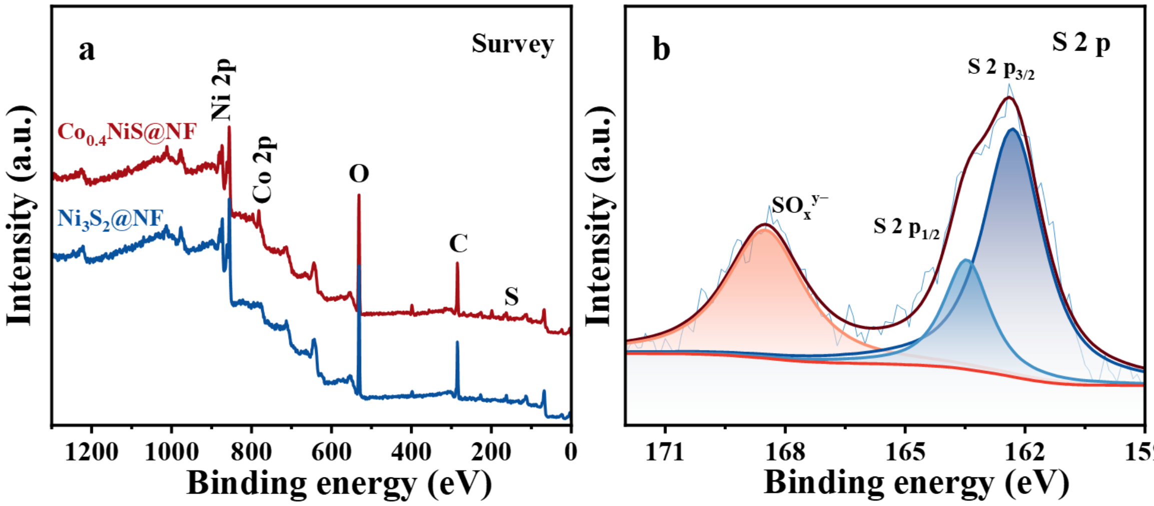

Figure S3. XPS survey spectra of a) $Co_{0.4}$NiS@NF and Ni_3S_2@NF, and b) high-resolution XPS spectra of S 2p for $Co_{0.4}$NiS@NF.

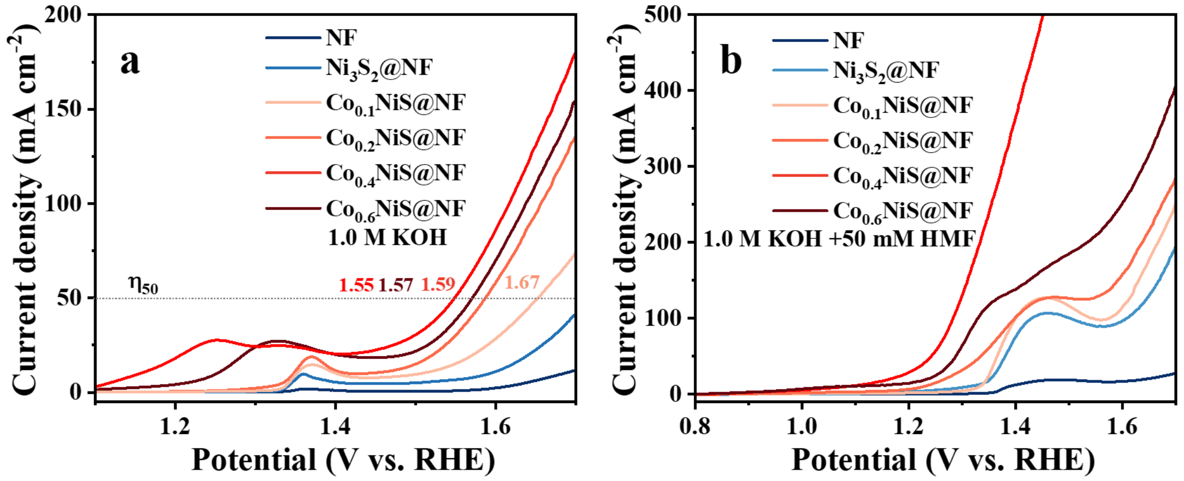

Figure S4. LSV curves (without IR compensation) for the NF, Ni_3S_2@NF and Co_xNiS@NF with various Co contents ($Co_{0.1}$NiS@NF, $Co_{0.2}$NiS@NF, $Co_{0.4}$NiS@NF, and $Co_{0.6}$NiS@NF) for a) OER and b) HMF electrooxidation.

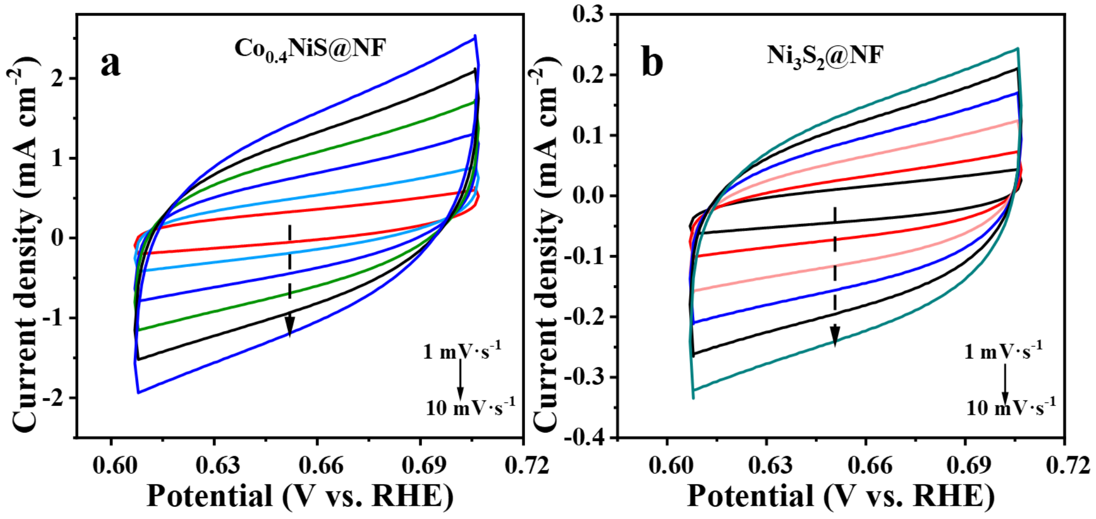

Figure S5. a) CV curves of $Co_{0.4}$NiS@NF and b) Ni_3S_2@NF with 50mM HMF at different scan rates.

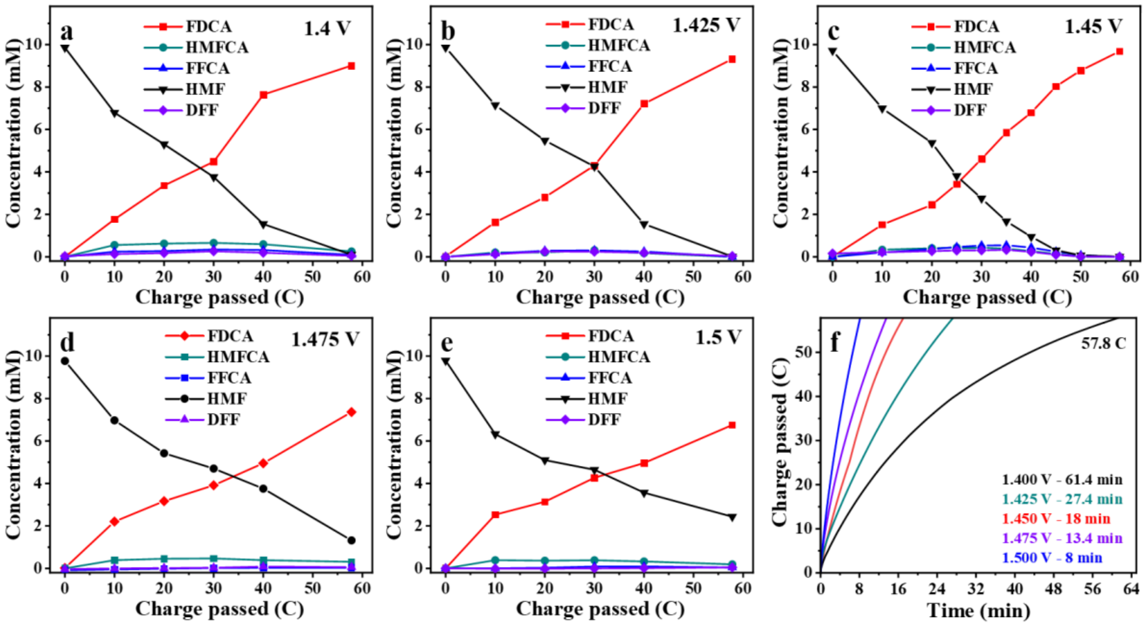

Figure S6. a-e) The concentrations of HMF, HMFCA, FFCA, HMF, DFF, and FDCA) with different electrolysis potentials under the fixed theoretical charge of 57.8 C. f) the time to reach theoretical charge (57.8 C) at different potentials.

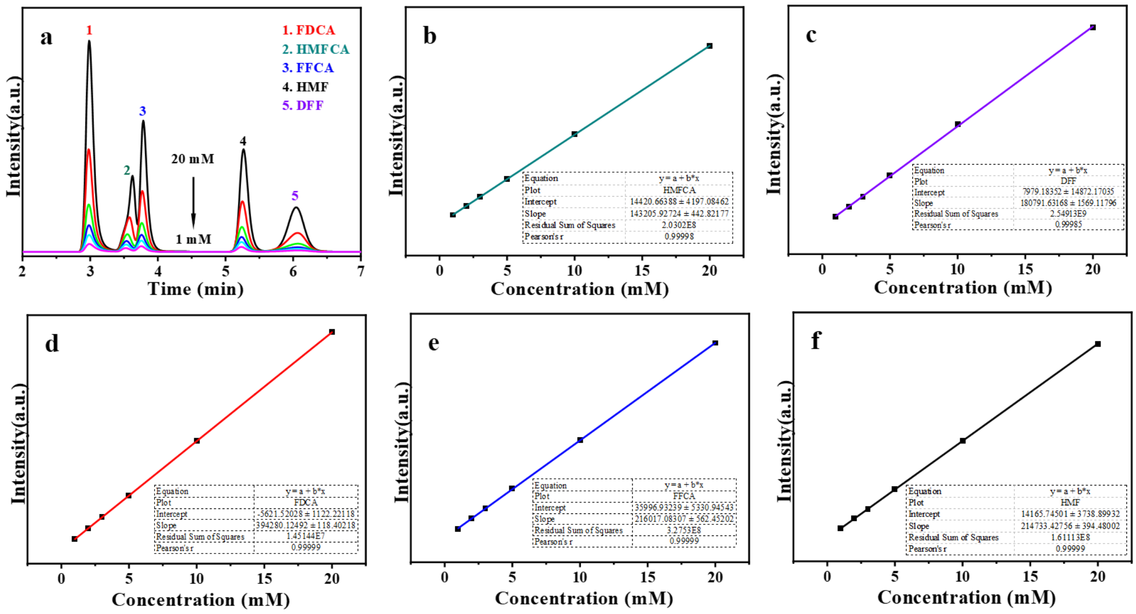

Figure S7. a) Reference HPLC spectra, and calibration curves for b) HMFCA, c) DFF, d) FDCA, e) FFCA, and (f) HMF.

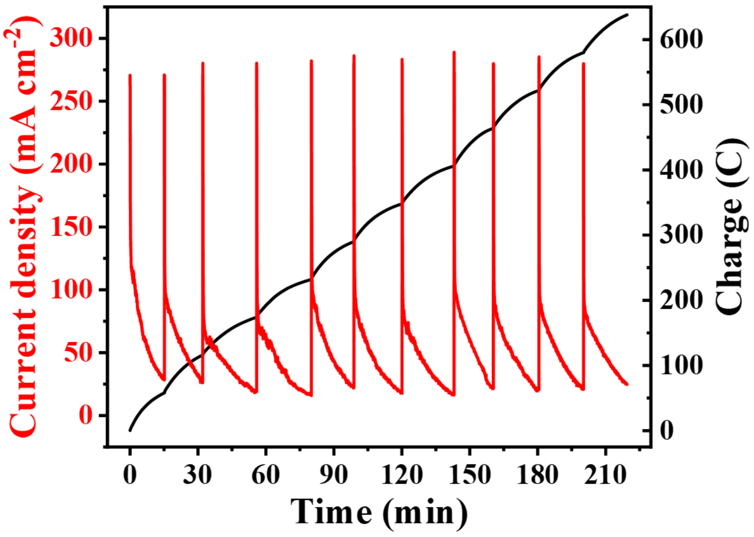

Figure S8. The variations of current and charge versus the electrooxidation time for eleven consecutive cycles of HMF electrooxidation by replacing the electrolyte. At each cycle, the total charge is set at 57.8 C.

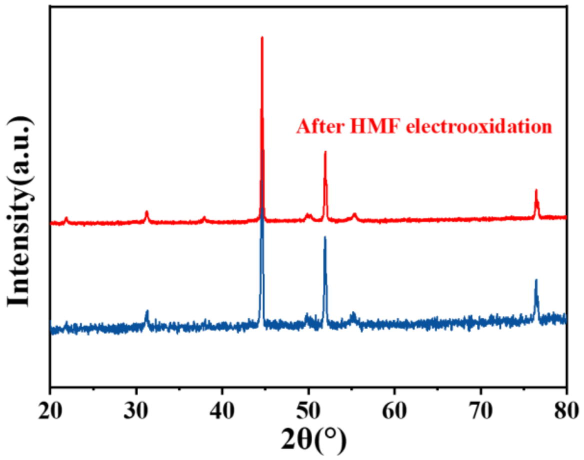

Figure S9. XRD patterns of the as-synthesized $Co_{0.4}NiS$@NF before and after HMF electrooxidation.

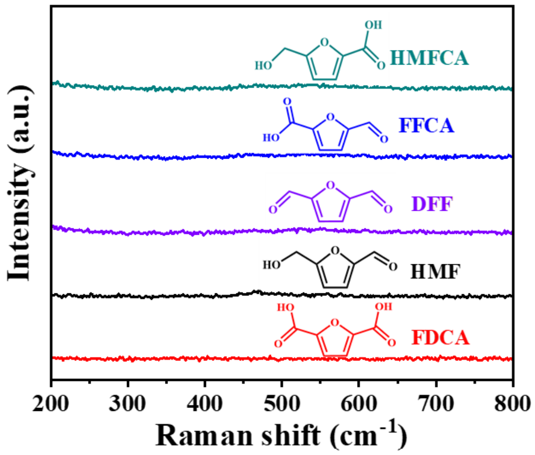

Figure S10 . Raman spectra of HMF, FDCA, DFF, FFCA and HMFCA.

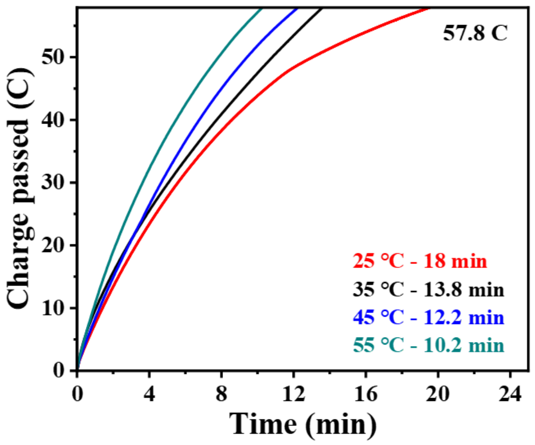

Figure S11. The reaction time to reach theoretical charge (57.8 C) at different electrolysis temperatures.

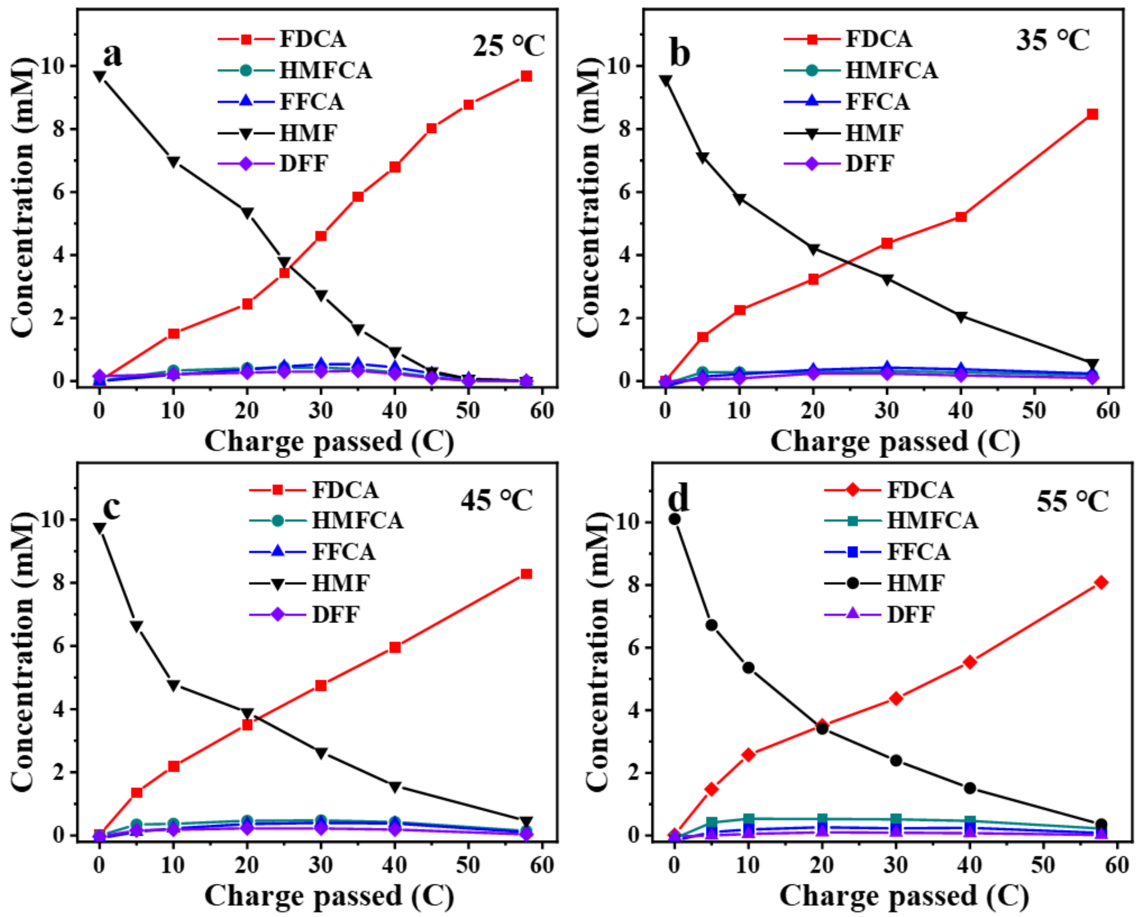

Figure S12. a-d) The concentration changes of HMF and its oxidation products with different temperatures under the fixed theoretical charge of 57.8 C.

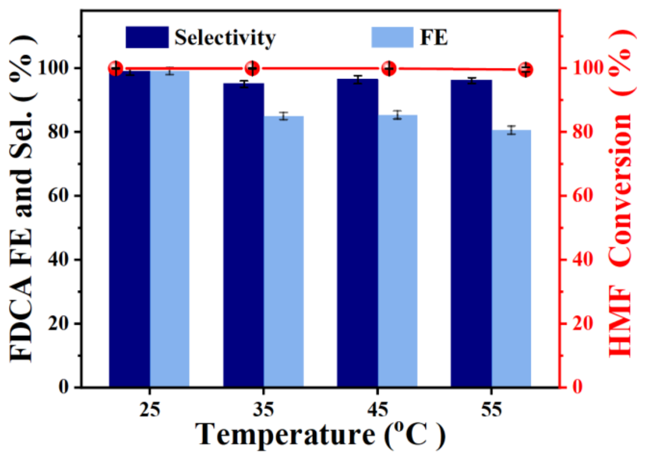

Figure S13. The FE and selectivity of the FDCA, and HMF conversion at different electrolysis temperatures.

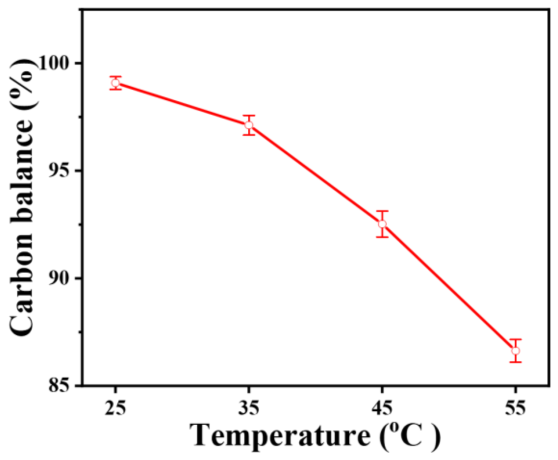

Figure S14. The carbon balances at different electrolysis temperatures.

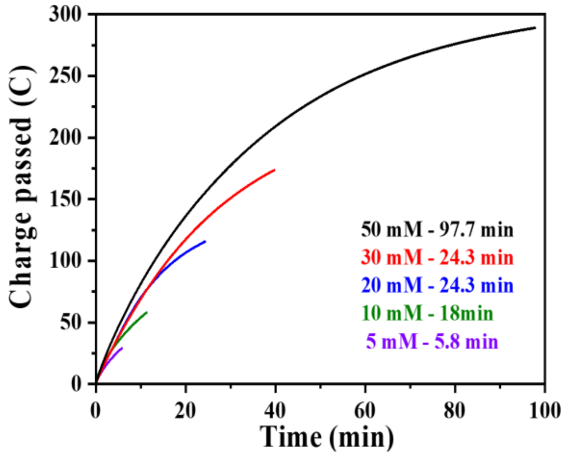

Figure S15. The time required to reach theoretical charge with different concentrations of HMF.

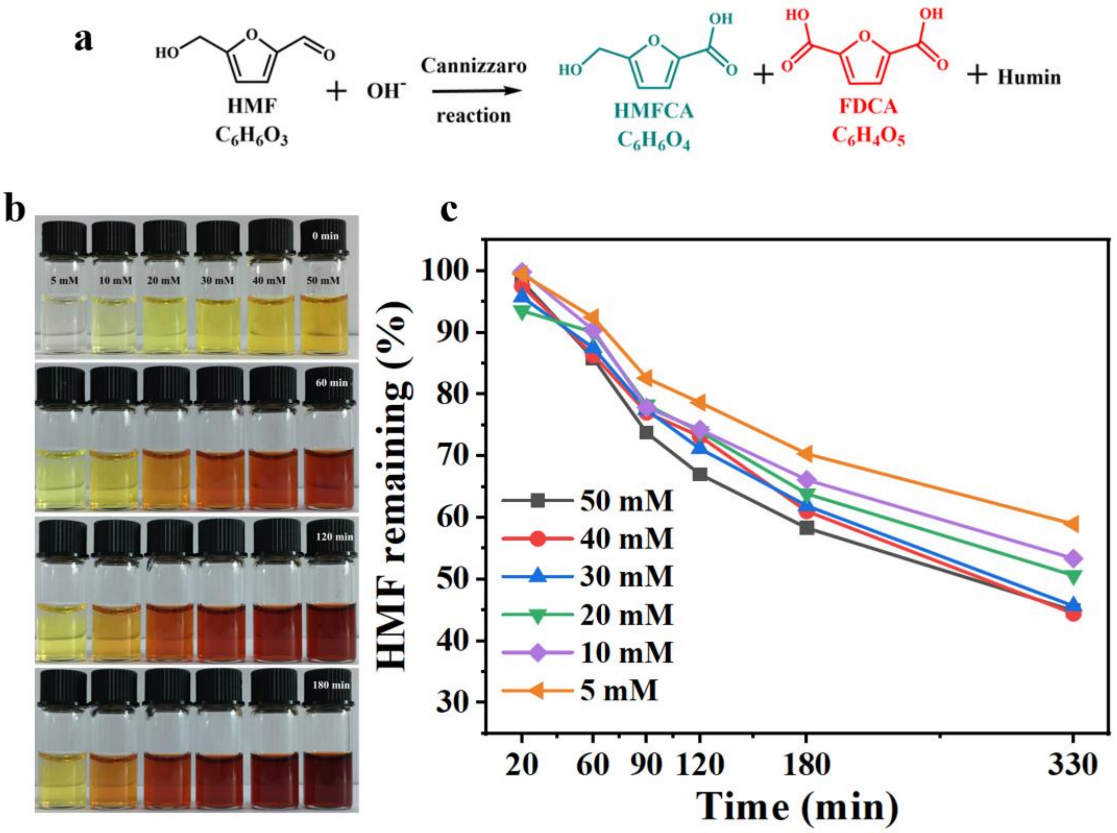

Figure S16. a) Possible HMF self-degradation mechanism due to the Cannizzaro reaction. b) The color changes and c) the stability tests of 5 ~ 50 mM of HMF in 1.0 M KOH at 25°C using $Co_{0.4}NiS$@NF electrocatalyst.

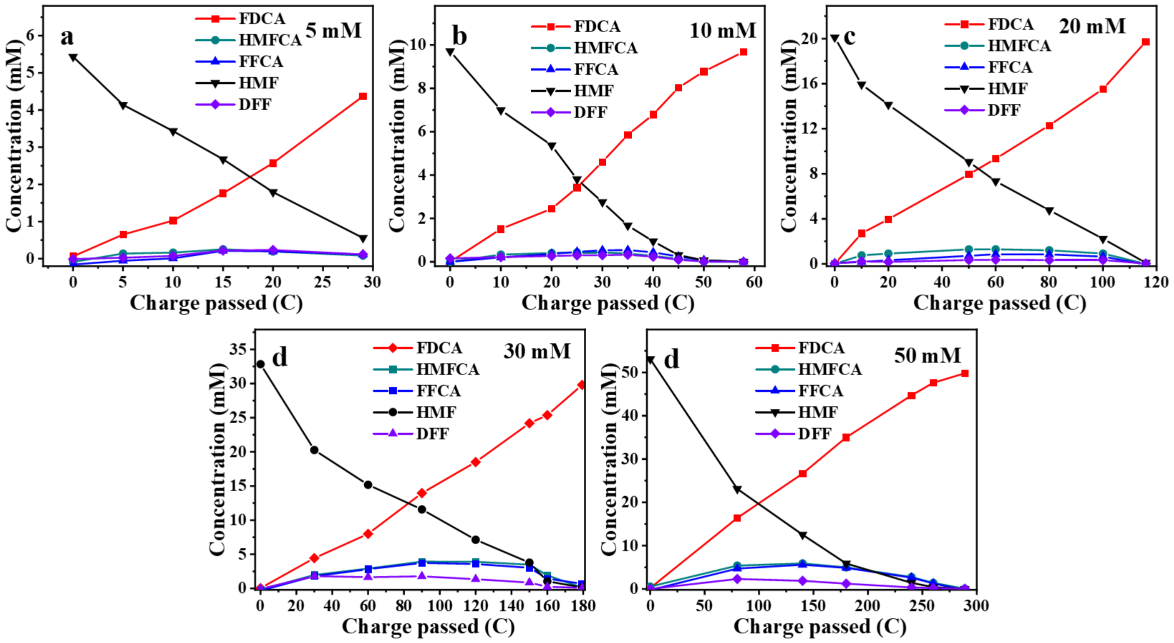

Figure S17. (a-e) the concentration of HMF, intermediates, and oxidation product changes during the HMF electrooxidation electrolysis.

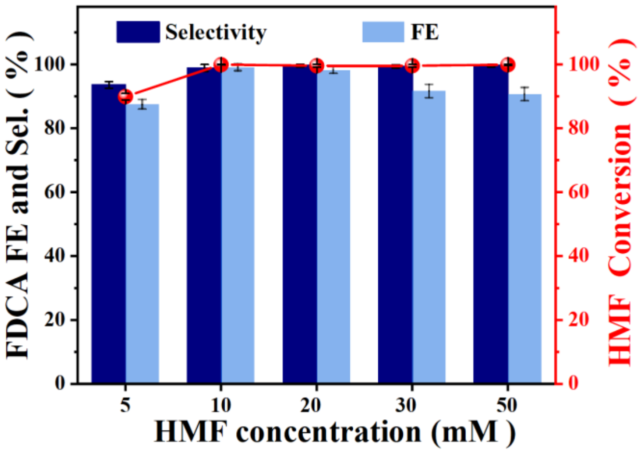

Figure S18. The FE and selectivity of the FDCA, and HMF conversion with different concentrations of HMF.

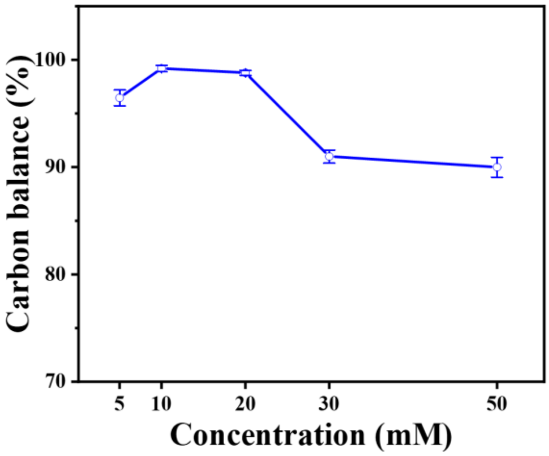

Figure S19. The carbon balances at different electrolysis temperatures.

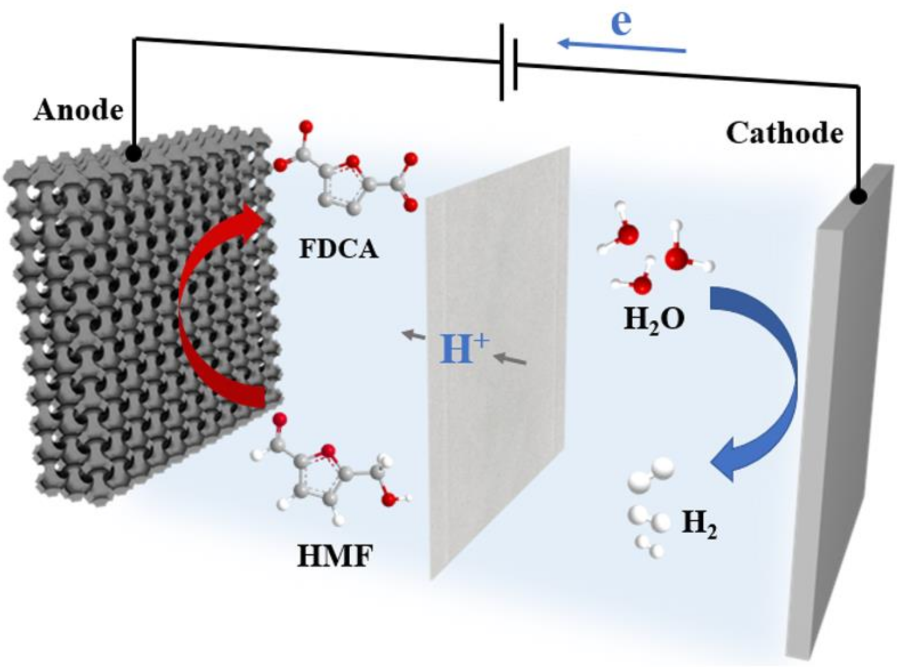

Figure S20. Schematic illustration of the paired electrolysis system for the HMF electrooxidation to FDCA and HER.

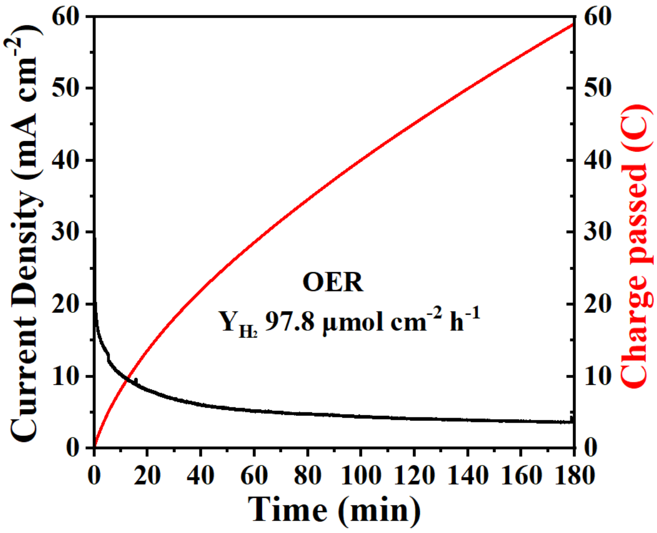

Figure S21. The current–time and charge–time curves during OER.

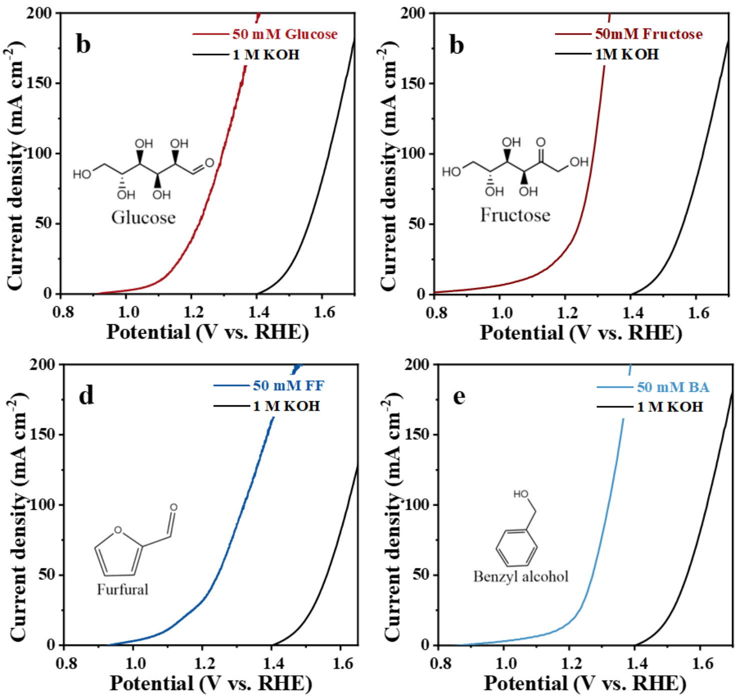

Figure S22. a-d) LSV curves of the $Co_{0.4}NiS$@NF at a scan rate of 5 mV s^{-1} in 1.0 M KOH with and without glucose, fructose, FF (furfural), and BA (benzyl alcohol).

Table S1. The ratio for Co-doped Ni_3S_2@NF with various Co content.

Sample	Co content [PPB]	Ni content [PPB]	Co/Ni	Amount of $CoCl_2 \cdot 6H_2O$ [mmol]
NiS@NF	0	590	0	0
$Co_{0.1}$NiS@NF	23.8	550.1	1/23.5	0.1
$Co_{0.2}$NiS@NF	64.7	461.1	1/7.1	0.2
$Co_{0.4}$NiS@NF	99.7	364.6	1/3.7	0.4
$Co_{0.6}$NiS@NF	140.2	342.6	1/2.4	0.6

*Note that Co-doped Ni_3S_2@NF were peeled off from Ni foam for ICP-AES characterizations.

Table S2. The comparison of activity for $Co_{0.4}$NiS@NF and other reported HMF electrochemical oxidation catalysts.

Electrocatalysts	Onset potential [V vs. RHE]	C_{HMF} [mM]	Electrolytic Time [min]	Con. [%]	Sel. [%]	FE [%]	Ref.
CoNiS@NF	0.9	10	18	100	100	99.1	Our work
Ir-Co_3O_4	1.15	50	-			98	[1]
Co_3O_4	-	5	-	99.6	90.8		[2]
CoO-$CoSe_2$	1.30	10	60	-	-	97.9	[3]
CoP/CF	1.30	50	360	100	90	-	[4]
CoOOH	~1	5	1320	95.5	-	35.1	[5]
Ni_3S_2/NF	1.423	10	300	100	-	-	[6]
NiCoFe-LDHs	1.55	10	60	95	95	-	[7]
NiO-Co_3O_4	1.28	10	-	-	-	96	[8]
Ni_3N	1.35	50	-	-	-	-	[9]
$Ni(OH)_2$/NF	-	10	90	100	-	99	[10]
NiFe LDH	1.25	100	600	99	-	99.4	[11]
NiOOH	~1.2	5	282	99.8	-	96	[5]
Branched Ni	1.39	-	-	-	-	-	[12]
Ni_2P/NF	1.35	10	-	100	100	-	[13]
NixB/NF	1.45	10	30	100	-	-	[14]
$NiCo_2O_4$/NF	1.43	5	53	99.6	90.8	-	[15]
Ni_3N-V_2O_3	-	10	112	-	98.7	-	[16]

Table S3. The comparison of current density for $Co_{0.4}$NiS@NF and other reported HMF electrochemical oxidation catalysts.

Electrocatalysts	Potential and current	C_{HMF} [mM]	Ref.
CoNiS@NF	1.35 V (244 mA cm^{-2})	50	Our work
CoNiS@NF	1.4 V (368 mA cm^{-2})	50	Our work
CoNiS@NF	1.45 V (497 mA cm^{-2})	50	Our work
$CuCo_2O_4$	1.37 V (150 mA cm^{-2})	50	[17]
CoP/CF	1.425 V (20 mA cm^{-2})	50	[4]
Pt/$Ni(OH)_2$	1.45 V (30 mA cm^{-2})	50	[18]
Ni/NiO	1.49 V (100 mA cm^{-2})	50	[19]
CoOOH	1.423 V (＞300 mA cm^{-2})	50	[20]
Ni_3N	1.45 V (＞10 mA cm^{-2})	50	[9]
Ir-Co_3O_4	1.45 V (＞10 mA cm^{-2})	50	[1]
NiB	1.45 V (100 mA cm^{-2})	10	[14]
NiFe LDH	1.43 V (100 mA cm^{-2})	10	[11]
Ni_2P	1.423 V (＞200 mA cm^{-2})	10	[13]
Ni_2S_3	1.423 V (＞200 mA cm^{-2})	10	[6]

Table S4. The FDCA yield rates on the $Co_{0.4}$NiS@NF catalyst under different potentials.

Potential [V vs. RHE]	1.4	1.425	1.45	1.475	1.5
FDCA yield rate [μmol cm^{-2} h^{-1}]	90.5	212.8	330.4	338.8	506.7

Table S5. The ratio of Ni^{3+}/Ni^{2+} and Co^{3+}/Co^{2+} was obtained from XPS results.

	Ni^{3+}/Ni^{2+}	Co^{3+}/Co^{2+}
Pristine	2.3	0.3
After HMF electrooxidation	0.3	1.3
After OER	4.1	2.4

Table S6. The FDCA yield rates on the $Co_{0.4}NiS$@NF catalyst under different HMF temperatures.

Temperature [℃]	25	35	45	55
FDCA yield rate [μmol cm^{-2} h^{-1}]	330.4	368.7	408.1	475.5

Table S7. The FDCA yield rates on the $Co_{0.4}NiS$@NF catalyst under different HMF concentrations.

Concentration [mM]	5	10	20	30	50
FDCA yield rate [μmol cm^{-2} h^{-1}]	456.1	330.4	488.3	412.6	276.4

References

[1] Y. Lu, T. Liu, C. L. Dong, Y. C. Huang, Y. Li, J. Chen, Y. Zou, S. Wang, *Adv. Mater.* **2021,** *33*, 2007056.

[2] M. J. Kang, H. Park, J. Jegal, S. Y. Hwang, Y. S. Kang, H. G. Cha, *Appl. Catal., B* **2019,** *242*, 85.

[3] X. Huang, J. L. Song, M. L. Hua, Z. B. Xie, S. S. Liu, T. B. Wu, G. Y. Yang, B. X. Han, *Green Chem.* **2020,** *22*, 843.

[4] N. Jiang, B. You, R. Boonstra, I. M. Terrero Rodriguez, Y. Sun, *ACS Energy Lett.* **2016,** *1*, 386.

[5] B. J. Taitt, D.-H. Nam, K.-S. Choi, *ACS Catal.* **2018,** *9*, 660.

[6] B. You, X. Liu, N. Jiang, Y. Sun, *J. Am. Chem. Soc.* **2016,** *138*, 13639.

[7] M. Zhang, Y. Q. Liu, B. Y. Liu, Z. Chen, H. Xu, K. Yan, *ACS Catal.* **2020,** *10*, 5179.

[8] Y. X. Lu, C. L. Dong, Y. C. Huang, Y. Q. Zou, Y. B. Liu, Y. Y. Li, N. N. Zhang, W. Chen, L. Zhou, H. Z. Lin, S. Y. Wang, *Sci. China Chem.* **2020,** *63*, 980.

[9] B. Zhou, C.-L. Dong, Y.-C. Huang, N. Zhang, Y. Wu, Y. Lu, X. Yue, Z. Xiao, Y. Zou, S. Wang, *J. Energy Chem.* **2021,** *61*, 179.

[10] J. Zhang, W. Gong, H. Yin, D. Wang, Y. Zhang, H. Zhang, G. Wang, H. Zhao, *ChemSusChem* **2021,** *14*, 2935.

[11] W. J. Liu, L. N. Dang, Z. R. Xu, H. Q. Yu, S. Jin, G. W. Huber, *ACS Catal.* **2018,** *8*, 5533.

[12] A. R. Poerwoprajitno, L. Gloag, J. Watt, S. Cychy, S. Cheong, P. V. Kumar, T. M. Benedetti, C. Deng, K. H. Wu, C. E. Marjo, D. L. Huber, M. Muhler, J. J. Gooding, W. Schuhmann, D. W. Wang, R. D. Tilley, *Angew. Chem. Int. Ed.* **2020,** *59*, 15487.

[13] B. You, N. Jiang, X. Liu, Y. Sun, *Angew. Chem. Int. Ed.* **2016,** *55*, 9913.

[14] S. Barwe, J. Weidner, S. Cychy, D. M. Morales, S. Dieckhofer, D. Hiltrop, J. Masa, M. Muhler, W. Schuhmann, *Angew. Chem. Int. Ed.* **2018,** *57*, 11460.

[15] M. J. Kang, H. Park, J. Jegal, S. Y. Hwang, Y. S. Kang, H. G. Cha, *Appl. Catal., B* **2019,** *242*, 85.

[16] S. Liang, L. Pan, T. Thomas, B. Zhu, C. Chen, J. Zhang, H. Shen, J. Liu, M. Yang, *Chem. Eng. J.* **2021,** *415*, 128864.

[17] Y. Lu, C. L. Dong, Y. C. Huang, Y. Zou, Z. Liu, Y. Liu, Y. Li, N. He, J. Shi, S. Wang, *Angew. Chem. Int. Ed.* **2020,** *59*, 19215.

[18] B. Zhou, Y. Li, Y. Zou, W. Chen, W. Zhou, M. Song, Y. Wu, Y. Lu, J. Liu, Y. Wang, S. Wang, *Angew. Chem. Int. Ed.* **2021,** *60*, 22908.

[19] J. Wang, Z. Zhao, C. Shen, H. Liu, X. Pang, M. Gao, J. Mu, F. Cao, G. Li, *Catal. Sci. Technol.* **2021,** *11*, 2480.

[20] R. Zhang, S. Jiang, Y. Rao, S. Chen, Q. Yue, Y. Kang, *Green Chem.* **2021,** *23*, 2525.
